# Dermatoprotective Potential of *Hibiscus sabdariffa* L.: A Systematic Review of Preclinical Evidence

**DOI:** 10.3390/ijms27219473

**Published:** 2026-10-23

**Authors:** Olufemi Gabriel Abimbola, Oluseun Peter Bolawa, Mariáurea Matia Sarandy, Rômulo Dias Novaes, Reggiani Vilela Gonçalves, Maria Do Carmo Gouveia Peluzio

**Affiliations:** 1 Department of Nutrition and Health, Federal University of Viçosa, Viçosa 36570-900, MG, Brazil; olufemi.abimbola@ufv.br; 2 Department of Animal Biology, Federal University of Viçosa, Viçosa 36570-900, MG, Brazil; oluseun.bolawa@ufv.br; 3 Health Center, Biomedical Science Program, University of Vale do Rio Doce (UNIVALE), Governador Valadares 35020-220, MG, Brazil; mariaureasarandy@gmail.com; 4 Institute of Biomedical Sciences, Department of Structural Biology, Federal University of Alfenas, Alfenas 37130-001, MG, Brazil; romulo.novaes@unifal-mg.edu.br; 5 Department of Animal Science, Plants for Human Health Institute, North Carolina State University, North Carolina Research Campus, Kannapolis, NC 28081, USA

**Keywords:** *Hibiscus sabdariffa* L., inflammatory skin diseases, preclinical studies, systematic review, oxidative stress, wound healing, skin inflammation, dermatoprotection, NF-κB, Nrf2

## Abstract

Inflammatory and degenerative skin disorders are associated with persistent inflammation, oxidative stress, tissue dysfunction, and impaired wound healing. This systematic review synthesized preclinical evidence on the biological effects and molecular mechanisms of *Hibiscus sabdariffa* L. in inflammatory and degenerative skin disorders and was prospectively registered in PROSPERO (CRD420251013303). Eligible studies were original, peer-reviewed in vitro or in vivo investigations evaluating *H. sabdariffa* L. extracts, formulations, or isolated bioactive compounds and relevant dermatological outcomes; non-original publications, human studies, other *Hibiscus* species, and studies without appropriate controls were excluded. PubMed/MEDLINE, Scopus, Web of Science, and Embase were searched on 24 March 2025. Study selection, data extraction, and risk-of-bias assessment were performed independently by two reviewers, with methodological quality assessed using the SYRCLE Risk of Bias tool supplemented with criteria relevant to preclinical phytopharmacological research. Sixteen studies were included: nine in vitro, six in vivo, and one combined study. Because of substantial heterogeneity, results were synthesized narratively using mechanistic mapping and comparative assessment rather than meta-analysis. Anti-inflammatory effects were reported in 13 studies (81.3%), antioxidant effects in 12 (75.0%), and wound-healing/tissue-regenerative effects in 9 (56.3%); anti-fibrotic/photoaging effects occurred in 7 (43.8%), and anti-melanogenic activity in 4 (25.0%). Heterogeneity and methodological limitations restrict direct comparison and condition-specific conclusions. These findings indicate dermatoprotective potential, but standardized preclinical and clinical studies are warranted. Funding was provided by the Fundação de Amparo à Pesquisa do Estado de Minas Gerais (FAPEMIG), the Coordination for the Improvement of Higher Education Personnel (CAPES), and the National Council for Scientific and Technological Development (CNPq).

## 1. Introduction

Inflammatory and degenerative skin disorders, including atopic dermatitis, psoriasis, acne, contact dermatitis, photoaging, and other conditions associated with chronic cutaneous inflammation and tissue damage, represent a substantial public health burden worldwide [1,2]. These disorders are associated with pruritus, pain, visible skin changes, impaired quality of life, and considerable healthcare costs [3]. Although corticosteroids, immunosuppressive agents, and biologic therapies remain important treatment options, their long-term use may be limited by adverse effects, cost, and disease recurrence [4], prompting interest in therapies that target multiple pathogenic processes.

Inflammation and oxidative stress are closely interconnected in many skin disorders. Dysregulated immune responses promote the release of pro-inflammatory mediators, including tumor necrosis factor-α (TNF-α), interleukin (IL)-6, and IL-1β [5], while excessive reactive oxygen and nitrogen species (ROS/RNS) and impaired antioxidant defenses involving superoxide dismutase (SOD), catalase (CAT), and glutathione peroxidase (GPx) exacerbate tissue injury [6]. This interaction contributes to extracellular matrix degradation, impaired wound healing, photoaging, and disease progression [7,8]. Therefore, agents that modulate both inflammatory and oxidative pathways may have broad dermatological relevance.

Natural products are increasingly investigated as sources of bioactive compounds with potential dermatological applications [9]. *Hibiscus sabdariffa* L. (roselle) contains anthocyanins, flavonoids, phenolic acids, hibiscus acid, and other polyphenols associated with antioxidant and anti-inflammatory activities [10,11]. However, its biological effects may vary according to plant material, extraction method, phytochemical composition, and formulation, making systematic evaluation of the available evidence necessary [12].

Preclinical studies suggest that *H. sabdariffa* L. may influence several processes relevant to skin health, including inflammatory signaling, oxidative stress, wound healing, extracellular matrix remodeling, melanogenesis, and photoaging. Reported mechanisms include modulation of NF-κB, MAPK, and Nrf2 pathways; attenuation of oxidative injury; promotion of tissue repair; and regulation of pigmentation and skin barrier-related processes [13,14,15,16].

Despite these findings, the evidence remains fragmented. Differences in plant materials, extraction procedures, formulations, experimental models, treatment protocols, and outcome measures complicate comparisons among studies [16,17,18]. Previous reviews have summarized the general phytochemical and pharmacological properties of *H. sabdariffa* L. [9,10,14,19], but they have not systematically integrated the preclinical evidence on its dermatological effects across inflammatory, oxidative, wound-healing, fibrotic, photoaging, and pigmentation-related outcomes while evaluating the underlying molecular mechanisms and methodological characteristics of the studies.

Therefore, this systematic review aimed to synthesize the preclinical evidence regarding the dermatological effects of *H. sabdariffa* L. extracts, formulations, and bioactive compounds. We evaluated outcomes related to inflammation, oxidative stress, wound healing and tissue regeneration, extracellular matrix remodeling, fibrosis, photoaging, and melanogenesis while considering intervention characteristics, experimental models, and methodological quality. By integrating therapeutic and mechanistic findings, this review identifies areas of consistent evidence, highlights current limitations, and provides a foundation for future translational research on *H. sabdariffa*-based dermatological interventions.

## 2. Methods

### 2.1. PICO Framework

The review followed the Population, Intervention, Comparator, and Outcomes (PICO) framework. The population comprised preclinical in vitro and in vivo models of dermatologically relevant diseases and biological processes, including inflammation, oxidative stress, wound healing, tissue regeneration, fibrosis, photoaging, and pigmentation. Accordingly, melanoma-derived cell lines were eligible when used to investigate pigmentation-related outcomes, and keloid fibroblast models were eligible when used to investigate fibrosis-associated processes. Their inclusion was based on the biological outcomes examined rather than an intention to evaluate clinical treatment of melanoma or keloid disease. The intervention comprised *H. sabdariffa* L. extracts, fractions, formulations, and isolated or plant-derived bioactive constituents. Comparators included untreated or vehicle controls and conventional therapeutic agents where applicable. Outcomes included anti-inflammatory and antioxidant responses, wound healing, tissue regeneration, antifibrotic and photoaging-related effects, anti-melanogenic activity, cytotoxicity or cell viability, and associated molecular or biochemical endpoints.

### 2.2. Literature Search Strategy

This systematic review was conducted in accordance with the Preferred Reporting Items for Systematic Reviews and Meta-Analyses (PRISMA) 2020 guidelines [20] and followed a predefined protocol prospectively registered in the International Prospective Register of Systematic Reviews (PROSPERO; CRD420251013303). No amendments were made to the registered protocol. Comprehensive searches were conducted on 24 March 2025 in PubMed/MEDLINE, Scopus, Web of Science, and Embase. The search strategies combined controlled vocabulary (MeSH and Emtree) with free-text terms relating to *Hibiscus sabdariffa* L., inflammatory skin diseases, oxidative stress, wound healing, and other dermatological outcomes. Boolean operators (AND and OR), field tags, and database-specific syntax were applied. No restrictions on language or publication date were imposed. The complete database-specific search strategies are provided in Supplementary Table S1.

**Figure 1:**
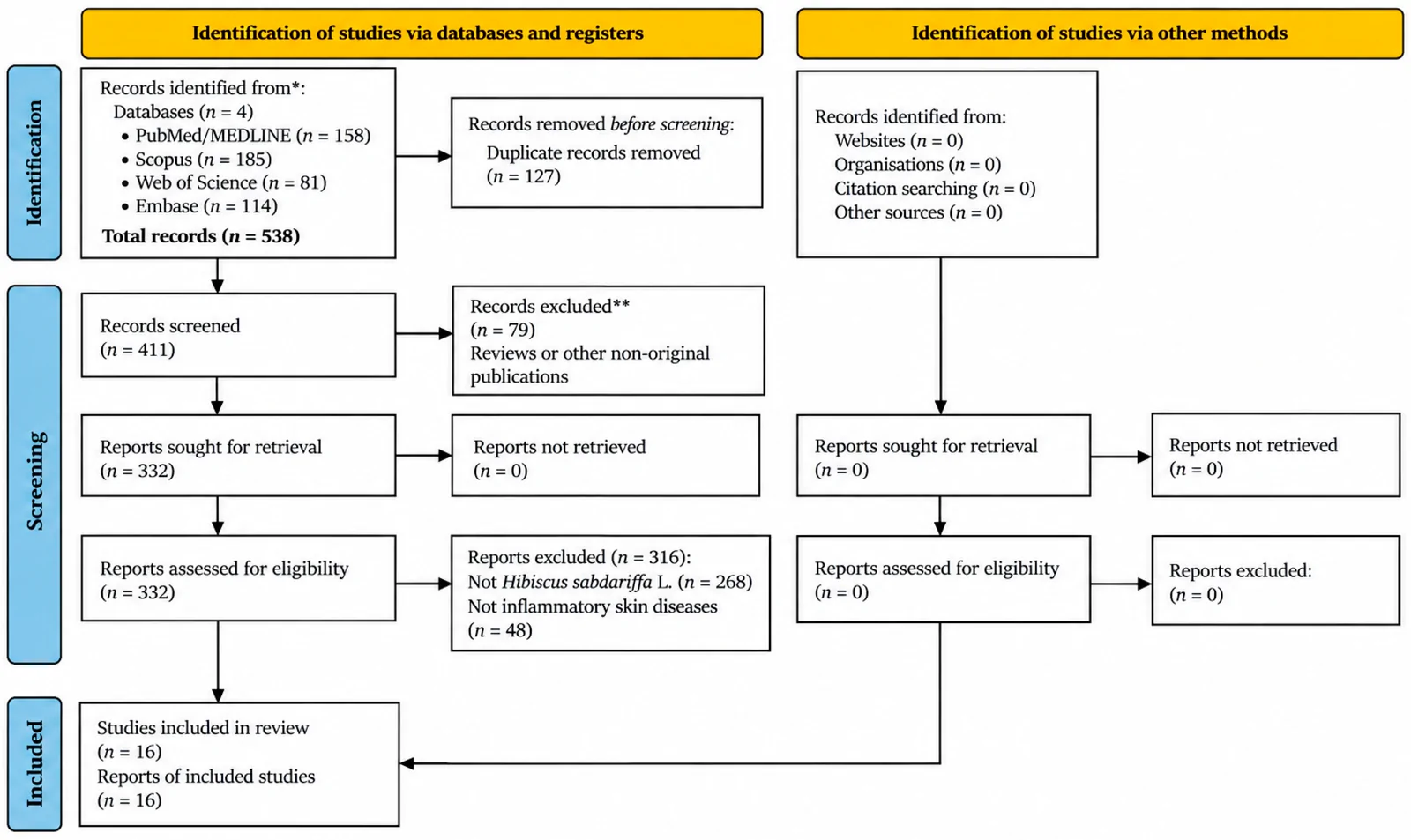
PRISMA 2020 flow diagram showing the identification, screening, eligibility assessment, and inclusion of studies in the systematic review. The diagram summarizes the records identified through databases and registers, the records removed before screening, the reports assessed for eligibility, and the studies included in the review. Yellow and blue boxes indicate the respective stages of the study-selection process. * Consider, if feasible to do so, reporting the number of records identified from each database or register searched (rather than the total number across all databases/registers). ** If automation tools were used, indicate how many records were excluded by a human and how many were excluded by automation tools [20].

After duplicate removal using EndNote 20 (Clarivate Analytics, Philadelphia, PA, USA), two reviewers (OPB and OGA) independently screened titles and abstracts and assessed the full texts of potentially eligible reports. Inter-reviewer agreement was assessed independently at both screening stages using Cohen’s kappa coefficient. The kappa value was 0.674 for title/abstract screening and 0.674 for full-text assessment. These values were calculated before consensus discussions and therefore represent agreement between reviewers before disagreements were resolved. Disagreements were subsequently resolved through discussion, with additional reviewers (MMS, MCGP, or RVG) consulted when necessary. Screening and eligibility assessments were conducted without automation tools. The numbers of records identified, screened, assessed for eligibility, excluded, and included are presented in the PRISMA flow diagram (Figure 1).

The search was not updated after 24 March 2025. Consequently, relevant studies published or indexed after this date may not have been captured, which should be considered when interpreting the completeness of the evidence included in this review.

### 2.3. Study Selection and Eligibility Criteria

Eligible studies were original, peer-reviewed experimental investigations using in vitro or in vivo preclinical models to evaluate *H. sabdariffa* L. extracts, formulations, fractions, or isolated bioactive compounds and at least one predefined dermatologically relevant outcome. Eligible outcomes included inflammatory responses, oxidative stress, wound healing and tissue regeneration, melanogenesis, fibrosis, photoaging, and associated cellular or molecular processes. Studies using melanoma-derived cells or keloid fibroblasts were eligible when their outcomes addressed these predefined domains; their inclusion did not imply that the models represented complete clinical disease phenotypes.

An appropriate control was defined as a comparator group that permitted interpretation of the intervention effect in the context of the experimental design and outcome assessed. Depending on the model and assay, eligible controls included untreated or vehicle-treated groups, baseline controls, disease- or injury-model controls, or other protocol-appropriate comparators. Positive controls receiving an established therapeutic agent or reference compound were considered supportive but were not mandatory when an adequate comparator was available. Studies were excluded when their design lacked a comparator sufficient to interpret the relevant outcome.

Combination interventions were eligible only when the contribution of *H. sabdariffa* L. could be distinguished from that of other active components. Studies were excluded when the effects attributable to *H. sabdariffa* L. could not be separated from those of another active ingredient. Studies investigating fractions, isolated constituents, or preparations derived from *H. sabdariffa* plant-cell cultures were eligible when they met the other inclusion criteria. However, their findings were interpreted in relation to the specific fraction or preparation investigated and were not assumed to represent the effects of conventional whole-plant or solvent-derived extracts.

Studies were excluded if they investigated *Hibiscus* species other than *H. sabdariffa* L.; involved human clinical participants; failed to assess any predefined outcome domain; or were reviews, editorials, conference abstracts, case reports, opinion articles, or other non-peer-reviewed publications.

Information on plant material, botanical identification or authentication, plant source, and voucher specimens was recorded when available in the original publications. Missing reports of authentication procedures or voucher specimens were treated as limitations in reporting and were not, by themselves, interpreted as evidence that botanical identification had not been performed.

Two reviewers (OGA and OPB) independently screened titles and abstracts and subsequently assessed potentially eligible reports in full text. Disagreements regarding eligibility were resolved through discussion with additional reviewers (MMS, RVG, or MCGP). The study selection process is presented in the PRISMA 2020 flow diagram (Figure 1).

### 2.4. Data Extraction and Management

Two reviewers (OGA and OPB) independently extracted data using a standardized form. Extracted information included study characteristics (authors, publication year, country, and journal); experimental characteristics (cell line or animal species, sex, age, weight, sample size, and ethical approval, where applicable); intervention characteristics (plant part, extraction method, phytochemical composition, dose, treatment duration, route, and comparator); and outcomes, including disease model, biomarkers, histopathological findings, molecular mechanisms, and biological effects. We resolved disagreements through discussion with additional reviewers (MMS, RVG, and MCGP) until consensus was reached. Relevant outcome measures, reported time points, and available analyses were extracted as described in the primary studies. When multiple compatible measures or time points were reported for the same outcome, the relevant reported results were considered for qualitative synthesis.

### 2.5. Data Synthesis

Given the methodological heterogeneity of the included studies, quantitative meta-analysis was not considered appropriate. Data were therefore synthesized using an integrative narrative approach combining qualitative evidence synthesis, structured mechanistic mapping, and comparative assessment of therapeutic outcomes.

**Effect measures.** No pooled effect estimates were calculated because of the substantial methodological and experimental heterogeneity among the included preclinical studies. Outcomes were synthesized using the biological endpoints and effect measures reported in the original studies, including biomarker concentrations, enzyme activities, gene expression, histopathological parameters, wound closure, cell viability, and related measures. Descriptive frequencies and percentages were calculated to summarize the number and proportion of included studies reporting each therapeutic outcome domain, using the 16 included studies as the denominator. These frequencies indicate how frequently an outcome domain was reported and do not represent effect sizes, reproducibility, therapeutic efficacy, or certainty of evidence. Outcome domains were not mutually exclusive because individual studies could investigate multiple biological processes. Where quantitative results were available, the reported direction of effects and statistical significance, where stated, were extracted and presented in the study-level tables. Effect estimates and measures of precision were neither recalculated nor pooled.

**Data conversion and handling.** No data conversions, statistical imputations, or cross-study normalization procedures were performed as part of the narrative synthesis. Missing or unclear information was recorded as not reported or not specified, where applicable. Potential sources of heterogeneity were explored qualitatively by comparing plant materials, extraction procedures, formulations, experimental models, treatment conditions, outcome measures, and methodological characteristics. Dose-related responses were considered within individual studies when multiple concentrations or administered doses were investigated; dose–response relationships were not assumed to be linear or directly comparable across different preparations and models. Animal sex was extracted where reported and considered as a potential source of heterogeneity. However, no formal sex-stratified synthesis was conducted, and sex-related differences were not inferred in the absence of direct comparisons. No sensitivity analyses were performed because quantitative meta-analysis or pooled statistical synthesis was not undertaken.

**Mechanistic evidence synthesis.** Mechanistic evidence was synthesized by mapping relationships among bioactive compounds, molecular targets, signaling pathways, biomarkers, and therapeutic outcomes. The synthesis distinguished direct molecular evidence from functional or downstream evidence and hypothesized relationships. Direct molecular evidence required measurement of a relevant pathway component or activation-associated endpoint, such as gene expression, protein abundance, phosphorylation, nuclear localization, or a pathway-specific functional assay. Functional or downstream evidence included changes in biochemical, cellular, or tissue outcomes consistent with a proposed pathway but insufficient to establish pathway activation or mediation. Hypothesized relationships were identified when a connection between a measured molecular response and a biological outcome was inferred but not directly tested. Gene-expression changes were not considered equivalent to protein-level activation, and downstream biomarker changes alone were not interpreted as proof of pathway-specific causality. Where reported, transcript-level, protein-level, and functional findings were distinguished to clarify the nature of the evidence supporting each proposed mechanism. Mechanistic interpretation also accounted for the experimental model, intervention characteristics, and consistency of findings across independent studies, without treating the number of studies or biomarkers alone as evidence of causal certainty.

**Mechanistic network construction.** A mechanistic network was constructed to summarize the relationships among major *H. sabdariffa* L. bioactive compounds and preparations, molecular targets, signaling pathways, biomarkers, and reported therapeutic outcomes. Directional relationships were assigned according to the direction of the measured change relative to the comparator reported in the original study. Solid arrows were reserved for directional relationships supported by direct measurements of the relevant molecular or pathway-associated endpoint, whereas dashed arrows represented hypothesized relationships that were biologically plausible or consistent with downstream findings but had not been directly tested. Where a pathway was not directly assessed, its involvement was identified as hypothesized rather than experimentally demonstrated. Arrow direction was not interpreted as proof that one measured change caused another, and the presence of a pathway in the network did not imply that it had been validated in every included study. Evidence was counted at the study level rather than by the number of biomarkers measured within an individual study. The network was therefore intended as a hypothesis-generating synthesis of reported mechanistic relationships rather than proof of causality or a unified mechanism of action.

**Assessment of therapeutic outcomes.** Therapeutic outcomes were grouped into five predefined domains: inflammatory response; oxidative stress; wound healing and tissue regeneration; anti-melanogenic activity; and anti-fibrotic/photoaging activity. Findings were qualitatively compared according to the direction and magnitude of reported effects where available, statistical significance, and consistency across biomarkers and experimental models. The synthesis also considered whether findings originated from in vitro or in vivo models and whether outcomes differed according to dose, endpoint, or experimental conditions. Favorable, mixed, null, or inconclusive findings were interpreted in the context of the comparisons and outcomes reported in the original studies. The frequency of studies reporting an outcome domain was not treated as evidence that all studies demonstrated a beneficial effect. Differences among experimental models, intervention preparations, and outcome measures were considered when interpreting the overall consistency and potential biological relevance of the findings.

### 2.6. Risk of Bias and Methodological Quality Assessment

Methodological quality and risk of bias were assessed using the SYRCLE Risk of Bias tool for animal intervention studies [21], supplemented with additional criteria relevant to preclinical phytopharmacological research. The SYRCLE domains were used to assess methodological features of the animal studies, while the supplementary criteria were used to evaluate aspects of intervention reporting and study quality relevant to botanical preparations and dermatological research.

The assessment considered randomization, allocation concealment, blinding, completeness of outcome data, selective reporting, and relevant baseline characteristics. Additional criteria covered botanical identification and extract characterization, extract purity and quality control, appropriateness of control groups, consistency of the intervention, ethical approval reporting, appropriateness of statistical methods, and applicability of the experimental findings to dermatological research.

Two reviewers (OGA and OPB) independently assessed each study. For each domain, judgments were classified as low, high, or unclear risk. Low risk was assigned when the available information supported adequate methodological practice for the domain assessed; high risk was assigned when the study report indicated a methodological limitation likely to introduce bias; and unclear risk was assigned when insufficient information was available to make a reliable judgment. For the supplementary criteria, the same principle was applied, taking into account the relevance of each criterion to the study design and intervention. Disagreements were resolved through discussion, with additional reviewers (MMS and RVG) consulted when necessary.

Ethical approval was assessed separately from the methodological risk-of-bias domains. In particular, failure to report ethical approval was distinguished from an explicit statement that approval had not been obtained. Similarly, insufficient information about extract characterization or purity was not automatically interpreted as evidence of high risk; such cases were classified according to the available evidence and the predefined assessment criteria.

Risk-of-bias judgments were summarized using traffic-light plots and methodological quality summaries generated in Review Manager (RevMan), version 5.4.1. Individual domain judgments and their supporting justifications are provided in Supplementary Table S2.

### 2.7. Assessment of Reporting Bias and Certainty of Evidence

**Reporting bias:** Publication bias and selective reporting were considered in interpreting the findings. Funnel plots and statistical tests for small-study effects were not performed because the included studies differed substantially in experimental models, interventions, outcomes, and outcome measures, and no meta-analysis was conducted. Consequently, the direction and magnitude of publication bias could not be estimated statistically. Potential reporting bias was considered qualitatively in relation to the predominance of published studies, the possibility that statistically significant or favorable findings were more likely to be disseminated, and the limitations identified during risk-of-bias assessment. The risk-of-bias assessment of individual studies was considered separately from publication bias across the evidence base.

**Certainty and strength of evidence.** A formal certainty-of-evidence framework, such as GRADE, was not applied because the evidence consisted exclusively of heterogeneous preclinical in vitro and in vivo studies, with substantial variation in biological models, interventions, experimental conditions, and outcome measures. These differences limited the comparability of results and did not support a common quantitative synthesis. Instead, an adapted qualitative assessment was used to interpret the evidence across outcome domains, considering the number of supporting studies, consistency and direction of findings, diversity and relevance of experimental models, methodological limitations and risk of bias, and the availability of directly measured biological or mechanistic outcomes. Study frequency was treated as a descriptive measure of how often an outcome was investigated or reported, rather than as a measure of effect magnitude, reproducibility, causal certainty, or overall evidence strength. No formal numerical certainty ratings were assigned.

## 3. Results and Discussion

### 3.1. Study Selection, Characteristics, and Experimental Models

The systematic search identified 538 records across PubMed/MEDLINE (*n* = 158), Scopus (*n* = 185), Web of Science (*n* = 81), and Embase (*n* = 114). After removal of 127 duplicate records, 411 records remained for title and abstract screening. Of these, 79 records were excluded as reviews or other non-original publications, leaving 332 reports for retrieval. All 332 reports were successfully retrieved and assessed for eligibility. Following application of the predefined eligibility criteria, 316 reports were excluded at the full-text stage, comprising 268 reports that did not concern *H. sabdariffa* L. and 48 that did not concern inflammatory or degenerative skin diseases. Consequently, 16 preclinical studies were included in the qualitative synthesis (Figure 1).

The characteristics of the included studies, including experimental models, *H. sabdariffa* L. preparations, treatment conditions, investigated outcomes, and principal findings, are presented in Table 1. Of the 16 studies, nine used in vitro models, six used in vivo models, and one combined both approaches. The in vitro investigations primarily employed human keratinocytes, fibroblasts, and melanoma cells, as well as murine macrophage and melanoma cell lines, to investigate inflammation, oxidative stress, wound healing, melanogenesis, cytoprotection, and fibrosis. Some studies also employed multicellular co-culture systems, providing more complex experimental settings for evaluating skin-related responses.

The in vivo studies predominantly employed Wistar rats, Wistar albino rats, and albino mice to investigate wound healing, burn injury, dermatitis, and photoaging. Most animal studies reported appropriate ethical approval for the experimental procedures. Collectively, the included studies encompassed a broad range of cellular and animal models representing different aspects of cutaneous inflammation, oxidative injury, tissue repair, pigmentation, fibrosis, and photoaging.

The diversity of experimental models reflects the broad range of biological effects investigated for *H. sabdariffa* L.; however, it also represents an important source of methodological heterogeneity within the evidence base. Differences in cell type, disease or injury model, experimental conditions, intervention characteristics, and outcome measurements limit direct comparison of findings across studies. Nevertheless, the combination of in vitro and in vivo evidence provides complementary information, with cellular models facilitating investigation of molecular and cellular responses and animal models providing additional evidence of tissue-level effects.

### 3.2. Geographical Distribution of Included Studies

**Figure 2:**
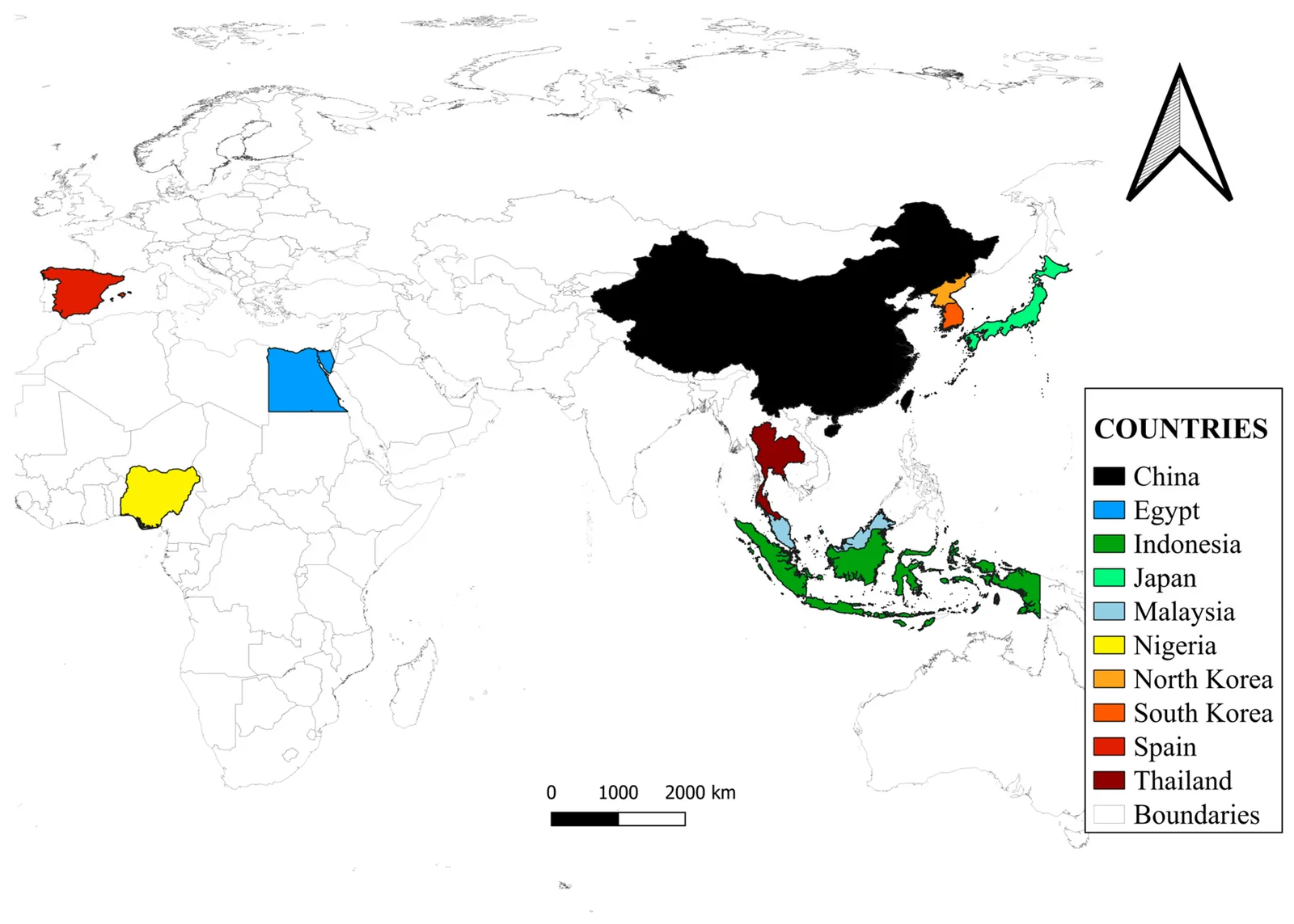
Geographical distribution of the included preclinical studies investigating the dermatological effects of *Hibiscus sabdariffa* L. published between 2005 and 2024. The map was generated using QGIS version 3.44.7 (QGIS Development Team; https://qgis.org/) based on the geographical locations of the studies included in this systematic review. Base map boundaries were obtained from the World Bank Open Data repository.

The 16 included studies originated from 10 countries across Asia, Africa, and Europe, with no studies conducted in the Americas (Figure 2). China and Indonesia each contributed three studies (18.8%), followed by Egypt and North Korea, with two studies each (12.5%). Japan, Malaysia, Nigeria, South Korea, Spain, and Thailand each contributed one study (6.3%). Overall, 14 studies (87.5%) originated from Asia and North Africa, whereas Europe and sub-Saharan Africa were less represented. This geographical distribution indicates that the current preclinical evidence base is concentrated in a limited number of regions, which may restrict the generalizability of findings across different research settings and experimental contexts.

**Geographical Distribution of Included Studies**

### 3.3. Intervention Characteristics of H. sabdariffa L.

The 16 included studies investigated diverse *Hibiscus sabdariffa* L. materials and preparations, including extracts from calyces, leaves, flowers, and fruits; solvent-derived fractions; formulated extracts; isolated constituents; and preparations derived from plant-cell cultures (Table 2; Supplementary Table S3). Extraction procedures included aqueous, ethanolic, methanolic, and hydroalcoholic methods, while formulations included creams, gels, ointments, and liposomal or nanoliposomal systems. The interventions also differed in dose or concentration, route of administration, and treatment duration. Their study-level characteristics, including phytochemical characterization where reported, are summarized in Table 2 and Supplementary Table S3.

The phytochemical constituents reported across the studies included anthocyanins, flavonoids, polyphenols, phenolic acids, hibiscus acid, and other individually identified compounds. However, the extent of phytochemical characterization varied considerably. Some studies reported broad classes of compounds, whereas others identified individual constituents or investigated enriched fractions and isolated compounds. Therefore, the presence of a reported constituent should be interpreted as a characteristic of the tested preparation rather than as definitive evidence that the constituent alone caused the observed biological response.

Certain studies investigated preparations that require distinction from conventional solvent-derived extracts. Kim et al. (2023) investigated Gly-Pro, a dipeptide derived from *H. sabdariffa*-related plant-cell material, and examined its effects on TGF-β1-induced collagen-related responses and the associated ATF4–serine/glycine biosynthesis pathway in human dermal fibroblasts [22] . These findings relate specifically to the tested Gly-Pro preparation and should not be generalized to conventional *H. sabdariffa* L. extracts. Similarly, Kim et al. (2024) evaluated polydeoxyribonucleotide derived from *H. sabdariffa* L. callus culture (HSC-PDRN) in cellular models relevant to wound healing and skin regeneration [23] . As a callus-derived DNA preparation, HSC-PDRN should be distinguished from crude extracts and solvent-derived fractions. Wang et al. (2022) investigated aqueous and ethanolic extracts, fractions, and the isolated constituent hibiscus acid; consequently, findings obtained with the isolated compound should be distinguished from those obtained with the more complex preparations [24].

Formulation was another source of variation among the interventions. For example, findings obtained with a nanoliposomal extract reflect the combined effects of the tested extract and its delivery system. Unless appropriate formulation controls were included, the effects cannot necessarily be attributed independently to the delivery system or to a specific phytochemical constituent. Similarly, where a preparation contained multiple components, attribution of the observed effects to *H. sabdariffa* L. was interpreted in relation to the comparators and experimental contrasts reported in the original study.

Botanical authentication and voucher-specimen information were not reported uniformly. Table 2 summarizes the authentication procedures and voucher details documented in the included publications, distinguishing reported information from information not reported. Rambe et al. (2022) reported validation of the leaves by Herbarium Medanese, Universitas Sumatera Utara [25]; Shadhan and Bohari (2017) reported a Forest Research Institute Malaysia catalogue number for the plant material [26]; and Nassar et al. (2025) reported authentication by a taxonomy specialist [27]. These examples illustrate variation in botanical documentation and should not be interpreted as evidence that all studies applied equivalent authentication procedures.

Overall, the included studies differed in plant material, extraction procedures, formulation, dose or concentration, treatment duration, route of administration, and phytochemical characterization. This heterogeneity limits direct comparisons and generalization across preparations. Supplementary Table S4 provides a study-level cross-tabulation of the specific preparations investigated and the dermatological outcome domains evaluated. Together, Table 2 and Supplementary Tables S3 and S4 facilitate comparison of intervention characteristics and outcome coverage while preserving distinctions among conventional extracts, solvent-derived fractions, isolated constituents, specialized formulations, and plant-cell-derived preparations.

### 3.4. Therapeutic Outcomes Across Biological Domains

Therapeutic outcomes reported across the 16 included studies are summarized in Table 3. Anti-inflammatory outcomes were reported in 13 studies (81.3%), antioxidant outcomes in 12 studies (75.0%), wound-healing and tissue-regenerative outcomes in nine studies (56.3%), anti-fibrotic/photoaging-related outcomes in seven studies (43.8%), and anti-melanogenic outcomes in four studies (25.0%). These percentages represent the proportions of included studies reporting each outcome domain, rather than measures of effect size, reproducibility, therapeutic efficacy, or certainty of evidence. The outcome domains were not mutually exclusive because individual studies investigated more than one biological process. Furthermore, the reporting of an outcome domain does not necessarily indicate that all endpoints evaluated within that domain showed statistically significant improvement.

**Anti-inflammatory and antioxidant outcomes.** Anti-inflammatory findings included reduced nitric oxide (NO) production and decreased levels or expression of TNF-α, IL-6, IL-1β, cyclooxygenase-2 (COX-2), and inducible nitric oxide synthase (iNOS). Antioxidant findings included reductions in reactive oxygen species (ROS) and malondialdehyde (MDA), alongside changes in superoxide dismutase (SOD), catalase (CAT), glutathione peroxidase (GPx), total antioxidant capacity (TAC), and glutathione-related measures. These outcomes were investigated in cellular models, including keratinocytes, fibroblasts, melanoma cells, and macrophages, while selected animal studies evaluated inflammatory or oxidative markers alongside tissue-level responses. The cellular studies provide evidence of biological responses under controlled experimental conditions, whereas animal studies provide additional information about responses within more complex biological systems. However, findings from these models cannot be considered directly equivalent.

Anti-inflammatory and antioxidant outcomes were frequently investigated within the same studies, suggesting a potential relationship between redox regulation and inflammatory signaling. Several studies also reported findings involving nuclear factor erythroid 2-related factor 2 (Nrf2)-associated signaling. Nevertheless, the available evidence does not establish a uniform causal relationship between antioxidant and anti-inflammatory activity because these responses were generally evaluated concurrently rather than through consistent pathway-specific intervention experiments. Differences in experimental conditions, intervention characteristics, and measured endpoints also limit direct comparisons across studies.

**Wound healing and tissue regeneration.** Wound-healing and tissue-regenerative outcomes were reported in nine studies (56.3%). In vitro studies investigated fibroblast migration and scratch-wound closure, whereas in vivo studies reported outcomes including wound contraction, re-epithelialization, collagen deposition, epithelial thickness, and hydroxyproline content. Additional molecular and cellular findings included modulation of collagen-related proteins, keratinocyte growth factor (KGF), vascular endothelial growth factor (VEGF), aquaporin-3 (AQP3), and extracellular matrix regulators. These findings are consistent with the coordinated roles of inflammation, cellular proliferation, extracellular matrix deposition, and tissue remodeling in cutaneous repair [28,29]. However, these outcomes represent different aspects of tissue repair and should not be interpreted as interchangeable measures of wound-healing efficacy. In particular, enhanced cellular migration or scratch-wound closure does not independently demonstrate complete tissue regeneration. Likewise, findings from animal burn and excision-wound models remain specific to the injury model, intervention, and treatment conditions investigated. Differences in dose, formulation, treatment duration, and outcome assessment prevent reliable comparisons of the relative efficacy of the preparations.

**Anti-fibrotic and photoaging-related outcomes.** Anti-fibrotic/photoaging-related outcomes were reported in seven studies (43.8%). Investigated endpoints included transforming growth factor beta 1 (TGF-β1), matrix metalloproteinases (MMPs), collagen architecture, hyaluronic acid content, and Bcl-2/Bax expression. Anti-fibrotic investigations included cellular fibroblast models examining collagen-related responses and signaling associated with TGF-β1/Smad2/3 and ATF4–serine/glycine biosynthesis. Photoaging-related investigations included UV-associated cellular or animal models in which oxidative damage, matrix-degrading activity, and extracellular matrix preservation were evaluated. Although these findings indicate biological activity in pathways relevant to fibrosis and photoaging, the diversity of models and endpoints prevents conclusions about a common magnitude of effect or a uniform mechanism of action across preparations.

**Anti-melanogenic activity.** Anti-melanogenic outcomes were reported in four studies (25.0%), primarily using melanoma-cell models. Reported endpoints included tyrosinase activity, intracellular melanin content, and microphthalmia-associated transcription factor (MITF) expression. These findings indicate potential modulation of melanogenesis under the experimental conditions investigated. However, results obtained from melanoma-cell models do not independently establish efficacy, safety, or suitability for treating pigmentation disorders in humans.

**Dose–response, sex, and methodological heterogeneity.** The included studies differed in the doses or concentrations tested, treatment durations, plant materials, extraction procedures, formulations, and experimental endpoints. Consequently, dose-related responses must be interpreted within the context of each individual study rather than extrapolated across preparations. Where multiple doses were examined, the response to a preparation could depend on the endpoint and experimental conditions; the available evidence does not establish a universal or linear dose–response relationship across the dermatological outcome domains. Animal sex was also reported inconsistently across the evidence base. Although sex was considered when reported in the extracted study characteristics, the available information does not support a reliable conclusion about whether sex modified the observed responses.

Overall, the included studies reported biological activity across inflammatory, oxidative, wound-healing, fibrotic, photoaging, and melanogenic domains. However, the frequency with which an outcome was reported should not be confused with the proportion of studies demonstrating a reproducible beneficial effect. The diversity of experimental models, intervention characteristics, outcome measures, and methodological reporting constrains assessment of consistency and comparative effects. The findings should therefore be interpreted as preliminary preclinical evidence of potential dermatoprotective activity rather than confirmation of therapeutic efficacy in humans.

### 3.5. Molecular Mechanisms Underlying the Dermatological Effects of H. sabdariffa L.

The molecular findings reported across the 16 included preclinical studies were synthesized according to the biological endpoints measured in each study. To avoid overstating mechanistic certainty, we distinguished direct measurements of pathway components from downstream biomarker changes and functional outcomes. Evidence included transcriptional changes, measurements of pathway-associated proteins or their phosphorylation, and biochemical, cellular, or tissue-level endpoints. However, changes in oxidative stress, inflammation, pigmentation, or tissue repair were not considered, by themselves, proof that a particular signaling pathway mediated the observed effect (Table 4).

**Nrf2-associated antioxidant responses.** Evidence related to nuclear factor erythroid 2-related factor 2 (Nrf2) was reported in studies investigating callus-derived polydeoxyribonucleotide (PDRN) and conventional *H. sabdariffa* L. preparations. Kim et al. (2024) [30] reported increased *Nrf2* transcript expression in human keratinocytes treated with *H. sabdariffa* L. callus-derived PDRN, alongside changes in antioxidant- and wound-repair-associated markers, including *SOD2*, *CAT*, *KGF*, *VEGF*, and *AQP3*. Kim et al. (2024) [23] These findings provide transcript-level evidence associated with Nrf2 and complementary protein-level evidence of antioxidant and repair-related responses. Nevertheless, increased *Nrf2* transcript expression does not establish Nrf2 nuclear translocation or transcriptional activity. Malar et al. (2022) [30] reported changes involving NRF2 and NQO1 alongside reduced oxidative injury in phenanthrene-exposed HaCaT keratinocytes. The precise interpretation of these findings depends on the molecular endpoints measured in the original study. Overall, the evidence supports an association between the tested preparations and cellular redox responses but does not establish that Nrf2 mediates all antioxidant effects reported for *H. sabdariffa* L.

**Inflammatory signaling and NF-κB-related mechanisms.** Several studies reported changes in inflammatory endpoints, including nitric oxide, pro-inflammatory cytokines, and other inflammatory mediators. Khalil et al. (2022) [31] for example, reported reduced TLR4, TNF-α, and IL-6 in a rat burn-wound model following treatment with an ethanolic flower extract. Wang et al. (2022) [24] also evaluated inflammatory responses in cellular models, including RAW 264.7 macrophages. These findings support modulation of inflammatory responses by the specific preparations tested. However, changes in inflammatory mediators or TLR4 expression alone do not demonstrate direct inhibition of nuclear factor kappa B (NF-κB). Unless a study directly measured NF-κB activation through endpoints such as p65 phosphorylation, nuclear translocation, DNA binding, or a reporter assay, the connection between treatment and NF-κB should be interpreted as a proposed mechanistic relationship rather than a demonstrated pathway effect.

**Figure 3:**
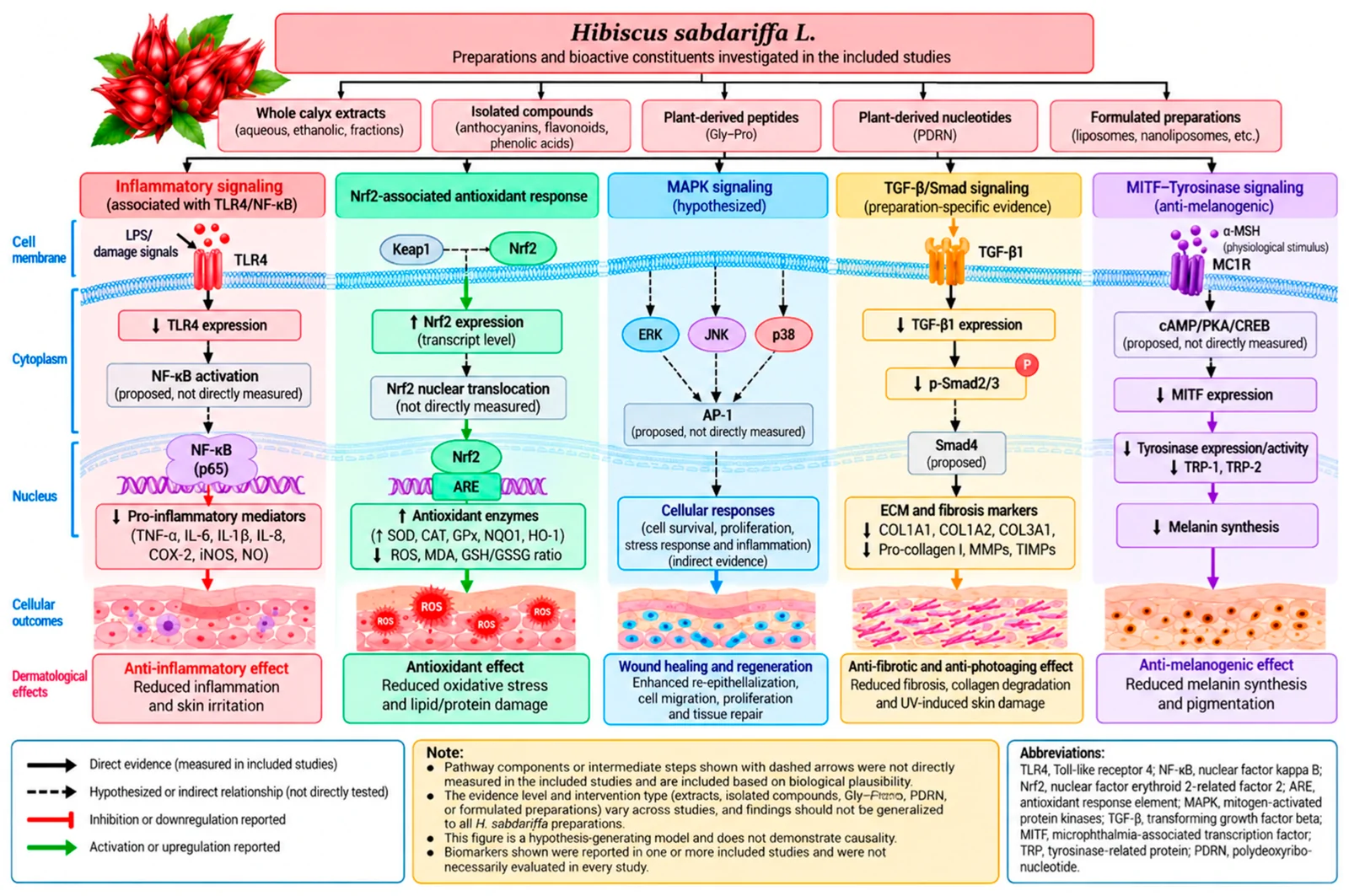
Hypothesis-generating mechanistic pathways underlying the dermatological effects of *Hibiscus sabdariffa* L. The diagram summarizes reported associations between *H. sabdariffa* L. preparations and bioactive constituents, pathway-associated molecular targets, biomarkers, and dermatological outcomes across the included preclinical studies. Solid arrows indicate relationships supported by direct measurements of the relevant molecular or pathway-associated endpoints, whereas dashed arrows indicate hypothesized or indirectly inferred relationships that were not directly tested. Red inhibitory symbols indicate reported inhibition or downregulation, and green arrows indicate reported activation or upregulation. These symbols represent the direction of reported changes and do not, by themselves, establish causal relationships. Transcript-level, protein-level, and functional evidence are distinguished where reported. Dashed pathway components, including proposed intermediate signaling steps, represent biologically plausible mechanisms rather than experimentally confirmed events. The evidence applies to the specific extracts, isolated compounds, plant-derived biomolecules, or formulated preparations investigated and should not be generalized to all *H. sabdariffa* L. preparations. Biomarkers shown were reported in one or more included studies and were not necessarily evaluated in every study. The figure is intended to generate mechanistic hypotheses rather than demonstrate causality or establish a unified mechanism of action. Source: Prepared by the authors using BioRender, Canva (web application), and Microsoft PowerPoint 2016 MSO (Version 2508, Build 16.0.19127.20800; 64-bit) on Windows 10, based on evidence synthesized from the included studies.

**MAPK signaling.** MAPK-related relationships should be considered experimentally supported only where an included study directly measured a relevant component or activation endpoint, such as ERK, JNK, or p38 phosphorylation, or employed a pathway-specific functional intervention. General changes in inflammation, oxidative stress, apoptosis, or cell proliferation are insufficient to establish MAPK modulation because these outcomes can arise through multiple signaling pathways. Accordingly, MAPK connections in Figure 3 that lack direct study-level support should be identified as hypothesized or omitted from the experimentally supported network.

**TGF-β/Smad signaling and extracellular matrix regulation.** More direct pathway-associated evidence was reported by Kim et al. (2023) [22] who investigated a Gly–Pro dipeptide preparation derived from plant-cell material in TGF-β1-stimulated human dermal fibroblasts. The study reported reductions in phosphorylated Smad2/3 and collagen-related markers, including COL1A1, COL1A2, COL3A1, and procollagen I, alongside changes in ATF4 and markers associated with serine and glycine biosynthesis. These findings support an effect of the tested Gly–Pro preparation on TGF-β1/Smad2/3-associated responses under the experimental conditions. Khalil et al. (2022) [31] reported reduced TGF-β in a rat burn-wound model, while Putra et al. (2020) [32] reported reduced TGF-β1 and changes in fibroblast responses in a human keloid-fibroblast model. These latter findings support changes in fibrosis-associated endpoints but do not independently establish direct inhibition of Smad signaling. The findings should therefore be interpreted in relation to the specific preparations and models investigated rather than generalized to all *H. sabdariffa* L. extracts.

**MITF–tyrosinase signaling and melanogenesis.** Evidence for anti-melanogenic activity came primarily from melanoma or melanocyte-related cellular models. Hwang et al. (2013) [33] reported reduced tyrosinase activity and protein expression, reduced microphthalmia-associated transcription factor (MITF) protein, and lower melanin content following treatment with liposome-encapsulated calyx anthocyanins. Li et al. (2020) [34] reported changes in MITF, tyrosinase, and tyrosinase-related proteins in a UVB-associated photoaging and pigmentation model. Cho et al. (2023) [17] also investigated melanogenesis alongside oxidative stress and skin-barrier-related outcomes. These findings support effects on melanogenesis-associated endpoints in the models tested. However, the MITF–tyrosinase axis should be presented as directly supported only to the extent that the relevant components and treatment-associated changes were measured. Changes in melanin content alone do not demonstrate that this pathway mediated the response.

**Causal inference, intervention specificity, and formulation effects.** Although several studies reported molecular changes consistent with antioxidant, anti-inflammatory, antifibrotic, or antimelanogenic responses, pathway-associated changes do not, by themselves, establish causal mediation. Stronger causal inference requires approaches such as pathway-specific pharmacological inhibition, gene knockdown or knockout, or rescue experiments demonstrating whether interruption or restoration of a pathway alters the response to treatment. The use of these approaches must be established from the original studies before a pathway is described as causally involved. Where causal experiments are not documented, the findings should be described as associated with, consistent with, or potentially involving the proposed pathway rather than demonstrating that the pathway is necessary or sufficient for the observed effect.

The identity of the intervention is also important. Crude extracts and phytochemical fractions contain multiple constituents that may act through overlapping or distinct biological processes, whereas isolated compounds and plant-derived biomolecules represent more specific interventions. For example, the findings reported for Gly–Pro and callus-derived PDRN support biological effects of these particular preparations; they cannot automatically be generalized to calyx extracts or other whole-plant preparations. Similarly, effects observed with liposome-encapsulated anthocyanins or nanoliposomal formulations may reflect contributions from both the active material and the delivery system. Without appropriate comparisons among the unformulated preparation, the formulated preparation, and the vehicle or carrier alone, their respective contributions cannot be separated confidently.

Potential off-target effects and interactions among phytochemicals introduce further uncertainty. Constituents within a complex extract may interact additively, synergistically, or antagonistically, and their effects may differ from those of isolated compounds. In addition, concentrations effective in cultured cells may not correspond to concentrations achievable in human skin following topical application or systemic administration. Differences in extraction procedures, chemical composition, dose, exposure duration, delivery system, and experimental model limit direct comparisons across studies. Because these factors were not uniformly evaluated in the included evidence, their influence remains uncertain and should not be treated as experimentally demonstrated.

Taken together, the included studies suggest that *H. sabdariffa* L-derived preparations may influence redox regulation, inflammatory responses, extracellular matrix remodeling, fibrosis-associated signaling, and melanogenesis. However, the level of evidence varies by pathway, intervention, and experimental model. Some studies measured specific pathway-associated transcripts or proteins, whereas others reported downstream biochemical, cellular, or tissue-level outcomes. The mechanistic network in Figure 3 is therefore presented as a hypothesis-generating synthesis of preparation-specific findings, not as proof of causal pathways or a single unified pharmacological mechanism. Directional arrows indicate the direction of measured changes relative to the study comparator, while connections that were not directly tested are identified as hypothesized.

### 3.6. Influence of Extraction Procedures and Formulation on Biological Activity

Differences in plant material and extraction procedures may contribute to variation in the phytochemical composition and biological activity of *H. sabdariffa* L. preparations. Solvent composition and polarity, extraction temperature, extraction time, and processing conditions can influence the recovery and relative abundance of anthocyanins, flavonoids, phenolic acids, and other constituents. Consequently, preparations derived from the same plant species may differ in their chemical profiles. However, the effects of individual extraction parameters depend on the specific conditions employed and cannot be inferred from the solvent category alone.

Formulation may introduce additional differences in the behavior of these preparations. Creams, gels, and ointments provide different delivery matrices, whereas liposomal and nanoliposomal systems may influence the protection, release, and delivery of incorporated compounds. These systems may offer advantages for selected constituents, but improvements in chemical stability, skin penetration, bioavailability, or biological activity must be demonstrated experimentally rather than assumed from the formulation type. Dose, administration route, exposure duration, and the biological model may also influence the observed outcomes.

Although the included studies investigated diverse extraction and formulation strategies, differences between separate studies do not establish the superiority of one solvent, extraction method, or formulation over another. Direct comparisons require sufficiently comparable experimental conditions, ideally involving the same plant material, standardized phytochemical characterization, comparable doses, and common biological endpoints. Where such comparisons were unavailable, the findings were interpreted as evidence of activity for the specific preparation tested rather than evidence that one preparation strategy was more effective than another.

Future research should therefore prioritize systematic comparisons of extraction conditions, quantitative phytochemical profiling, assessment of chemical stability, and evaluation of formulation performance using appropriate controls. Standardized reporting of botanical source, plant part, extraction conditions, formulation composition, dose, and treatment duration would improve reproducibility and help determine whether differences in biological outcomes reflect preparation characteristics or differences in experimental design.

### 3.7. Safety Assessment, Translational Relevance, and Generalizability

Safety and cytotoxicity. Safety evidence across the included studies was limited and inconsistently characterized. Several studies assessed cell viability or related endpoints in HaCaT keratinocytes, dermal fibroblasts, macrophages, and melanoma-derived cell lines. Wang et al. (2022) [24] investigated cellular viability alongside oxidative stress and inflammatory markers, while Shadhan and Bohari (2017) [26] evaluated fibroblast responses in an in vitro wound-healing model. Malar et al. (2022) [30] examined the protective effects of an ethanolic calyx extract against phenanthrene-induced toxicity in HaCaT keratinocytes. Although these findings provide information about cellular responses under the specific experimental conditions tested, they do not establish the overall safety of the preparations. The available safety and translational information is summarized in Supplementary Table S5.

Cell viability and cytoprotection should be distinguished from comprehensive toxicological assessment. A preparation that preserves cellular viability under one experimental condition may produce concentration-dependent or endpoint-specific adverse effects under other conditions. Likewise, apoptosis observed in melanoma-derived cells or keloid fibroblasts may represent the intended biological response in those experimental models and should not automatically be interpreted as toxicity to normal skin cells. Interpretation therefore requires consideration of cell type, concentration, exposure duration, comparator, and the specific endpoints measured.

The available evidence was insufficient to establish a comparative safety profile or a general LD_50_ value for *Hibiscus sabdariffa* L. preparations. Where acute or chronic toxicity, adverse effects, or LD_50_ values were not documented in the extracted evidence, these outcomes were treated as unavailable rather than as evidence of safety. Furthermore, the absence of reported adverse effects in a preclinical experiment does not demonstrate that a preparation is safe for human use. More systematic, concentration-dependent cytotoxicity testing and appropriately designed toxicological studies are needed to characterize safety across different preparations and routes of administration.

Bioavailability, skin penetration, and formulation effects. Translational interpretation is further complicated by the diversity of preparations and delivery systems investigated. The included studies examined conventional extracts, isolated constituents, plant-cell-derived biomolecules, and formulated preparations, including liposome-encapsulated anthocyanins and nanoliposomal extract formulations. Hwang et al. (2013) [33] investigated liposome-encapsulated anthocyanins in a melanogenesis model, whereas Nassar et al. (2025) [27] evaluated a nanoliposomal hydroalcoholic extract in an experimental skin-aging model. These studies illustrate the application of formulation strategies in preclinical dermatological research; however, biological activity following formulation does not, by itself, demonstrate enhanced skin penetration, systemic bioavailability, or improved pharmacokinetic performance.

Skin penetration, retention within relevant tissue compartments, formulation stability, and the enzymatic or microbial metabolism of the preparations were not consistently characterized in the evidence extracted for this review. Similarly, the available information on metabolite formation and biological activity was insufficient for a comparative assessment. It therefore remains uncertain whether concentrations effective in cultured cells can be achieved and maintained in the relevant skin layers following topical administration or through systemic exposure. Future studies should characterize skin permeation and retention, formulation stability, release kinetics, local and systemic exposure where relevant, metabolite formation, and exposure–response relationships. Appropriate vehicle controls and direct comparisons between formulated and non-formulated preparations are also necessary to distinguish the effects of the active material from those attributable to the delivery system.

Experimental models and generalizability. The 16 included studies comprised nine in vitro studies, six in vivo studies, and one study combining experimental approaches. The in vitro investigations used human keratinocytes (HaCaT), human dermal fibroblasts, human keloid fibroblasts, human melanoma-derived cells (A375), murine melanoma cells (B16F10 and B16F1), and murine macrophages (RAW264.7). These models enabled investigation of processes associated with oxidative stress, inflammation, pigmentation, fibrosis, apoptosis, and tissue repair. Nevertheless, immortalized cell lines, primary cells, and multicellular experimental systems differ in their biological characteristics and cannot fully reproduce the structural, immunological, metabolic, and barrier functions of intact human skin.

The animal studies predominantly employed rat models of excision wounds or burns, while mouse models were used to investigate UV-associated and chemically induced skin aging. Reported characteristics included female Wistar rats in Azizah et al. (2021) [35] male and female Wistar albino rats in Builders et al. (2013) [36], and male Wistar rats in Khalil et al. (2022) [31]. Animal sex and other experimental characteristics were not consistently reported across all studies, limiting assessment of biological variability and potential sex-related differences. Consequently, the findings should be interpreted in the context of the specific species, strains, sexes, disease or injury models, and experimental conditions investigated rather than generalized directly to human dermatological diseases.

Melanoma-derived cells and keloid fibroblasts were included because the predefined outcome domains encompassed pigmentation-related responses and fibrosis, respectively. Their inclusion does not imply that the review evaluated clinical treatment of melanoma or keloid disease. Melanoma-derived cells were used to investigate melanogenesis-associated endpoints and cellular responses to selected preparations, whereas keloid fibroblasts were used to examine fibrosis-associated processes. These models provide insight into selected cellular mechanisms but cannot independently establish therapeutic efficacy, disease selectivity, or safety in patients with melanoma or keloids.

Overall, the dermatological potential of *H. sabdariffa* L. remains promising but preliminary. Differences in plant material, extraction procedures, chemical composition, intervention type, dose, exposure duration, formulation, and experimental models limit direct comparisons across studies. The available evidence supports further investigation but is insufficient to establish clinically relevant dosing, a comprehensive safety profile, pharmacokinetic behavior, or therapeutic efficacy in humans. Future research should integrate standardized phytochemical characterization with concentration-dependent cytotoxicity testing, appropriate normal-cell controls, systematic toxicological assessment, formulation-specific exposure studies, and well-designed in vivo and clinical investigations.

**Table 1:** **Characteristics of the included preclinical models evaluating the dermatological effects of** ***H. sabdariffa*** **L.** The table summarizes the study type, experimental models, biological origin, cell types or animal strains, primary dermatological applications, and ethical approval status of the included preclinical studies.

S/N	Author (Year)	Study Type	Experimental Model	Biological Origin	Cell Type/Animal Strain	Main Application	Ethical Approval
1	Ticona et al. (2022) [15]	In vitro	Melanogenesis and inflammatory cell model	Murine	B16F10 melanoma cells and RAW 264.7 macrophages	Melanogenesis; inflammation	Not reported
2	Azizah et al. (2021) [35]	In vivo	Excision wound-healing model	Rat	Female Wistar rats (2.5–3 months; 150–250 g)	Wound healing	Approved by Universitas Brawijaya
3	Builders et al. (2013) [36]	In vivo	Excision wound-healing model	Rat	Male and female Wistar albino rats (180–200 g)	Wound healing	Animal Ethical Committee of NIPRD, Abuja, Nigeria
4	Chiu et al. (2015) [37]	In vitro	Melanogenesis model	Human	A375 melanoma cells	Anti-melanogenic activity	Not reported
5	Cho et al. (2023) [17]	In vitro	Keratinocyte melanocyte co-culture model	Human and murine	HaCaT keratinocytes and B16F1 melanoma cells	Inflammation; melanogenesis	Not specified
6	Hwang et al. (2013) [33]	In vitro	Melanogenesis model	Human	A375 melanoma cells	Anti-melanogenic activity	Not reported
7	Khalil et al. (2022) [31]	In vivo	Excision wound-healing model	Rat	Male Wistar rats (200–250 g)	Wound healing	Institutional Animal Care and Use Committee, FPDU (FPDU 10/2021)
8	Kim et al. (2023) [22]	In vitro	Fibrosis model	Human	Foreskin-derived human dermal fibroblasts (HDFs)	Anti-fibrotic activity	Not reported
9	Kim et al. (2024) [23]	In vitro	Keratinocyte–fibroblast culture model	Human	HaCaT keratinocytes and Detroit 551 fibroblasts	Cytoprotection; wound healing	Conducted under institutional guidelines and regulations
10	Li et al. (2020) [34]	In vivo	UVB-induced photoaging model	Mouse	Male mice (strain not specified)	Photoaging; oxidative stress	Not reported
11	Malar et al. (2022) [30]	In vitro	Keratinocyte oxidative stress model	Human	HaCaT keratinocytes	Oxidative stress; inflammation	Not reported
12	Nassar et al. (2025) [27]	In vivo	UV- and D-galactosamine-induced skin aging model	Mouse	Male albino mice (6 weeks old, 20–25 g)	Photoaging/skin aging	German University in Cairo Animal Ethics Committee (#PBL-2020-08)
13	Putra et al. (2020) [32]	In vitro	Fibrosis model	Human	Primary human keloid fibroblasts	Anti-fibrotic activity	Not reported
14	Rambe et al. (2022) [25]	In vivo	Excision wound-healing model	Rat	Male Wistar rats (150–200 g)	Wound healing and re-epithelialization	Universitas Sumatera Utara Ethics Committee (Protocol No. 356/KEP/USU/2021)
15	Shadhan and Bohari (2017) [26]	In vitro	Fibroblast culture model	Human	HSF 1184 skin fibroblasts	Wound healing	Not reported
16	Wang et al. (2022) [24]	In vitro	Multi-cell inflammatory skin model	Human and murine	NHDF-Ad fibroblasts, HaCaT keratinocytes, and RAW 264.7 macrophages	Oxidative stress; inflammation	Not reported

**Table 2:** **Intervention characteristics of** ***H. sabdariffa*** **L. evaluated in preclinical models of inflammatory and degenerative skin disorders.** The table summarizes the experimental models, dermatological conditions investigated, plant parts utilized, extract or formulation types, and major bioactive compounds identified across the included studies.

S/N	Author (Year)	Experimental Model and Skin Condition	Plant Material and Intervention	Major Bioactive Compounds	Controls and Botanical Authentication
1	Ticona et al. (2022) [15]	B16F10 murine melanoma cells and RAW 264.7 macrophages; melanogenesis and skin inflammation	Leaves; aqueous extract (AEHS)	Phenolic acids, flavonoids, anthocyanins	**Controls:** Kojic acid for melanogenesis; L-NMMA for nitric oxide-related assessment. **Authentication:** Department of Botany, Faculty of Pharmacy, Universidad Complutense de Madrid; accession No. 136081.
2	Azizah et al. (2021) [35]	Female Wistar rats with third-degree burn wounds; burn wound healing	Calyx; methanolic–ethanolic extract administered orally and topically	Anthocyanins, vitamin C, citric acid, malic acid, proteins	**Controls:** Cold-cream base and silver sulfadiazine. **Authentication:** Botanical authentication and voucher specimen not reported in the article.
3	Builders et al. (2013) [36]	Wistar albino rats with excision wounds; wound healing	Calyces; methanolic extract formulated as a cream	Flavonoids, phenolic compounds, anthocyanins, ascorbic acid, trace minerals	**Controls:** Blank cream/ointment and gentamicin comparator groups. **Authentication**: Identified by an ethnobotanist at NIPRD, Nigeria; voucher No. NIPRD/H/6328.
4	Chiu et al. (2015) [37]	Human A375 and B16F10 melanoma cells and WS1 fibroblasts; melanoma-related cellular responses	Leaves; polyphenolic leaf extract (HLP)	Epicatechin gallate, epigallocatechin, ellagic acid, catechin, quercetin, ferulic acid, protocatechuic acid	**Controls:** Exact untreated, vehicle and assay-specific comparators should be confirmed in the original article. **Authentication:** Botanical authentication and voucher specimen not reported in the article.
5	Cho et al. (2023) [17]	HaCaT keratinocytes and B16F1 melanoma cells; oxidative stress, melanogenesis and skin-barrier dysfunction	Leaves and callus cells; hot-water extracts (HSPE and HSCE)	Guanine, phenylalanine, tryptophan, adenine, uridine, adenosine, guanosine	**Controls:** Distilled water; kojic acid for melanin-related assays; N-acetyl-L-cysteine for antioxidant assessment; glyceryl glucoside for barrier-related assessment. **Authentication:** Voucher specimen not reported in the article.
6	Hwang et al. (2013) [33]	Human A375 melanoma cells; hyperpigmentation and melanogenesis	Calyx-derived anthocyanins encapsulated in liposomes	Anthocyanins	**Controls:** Non-encapsulated anthocyanins and formulation/assay controls, as applicable. **Authentication:** Botanical authentication and voucher specimen not reported in the article.
7	Khalil et al. (2022) [31]	Male Wistar rats with burn wounds; burn wound healing	Flowers; ethanolic extract formulated as an ointment	Anthocyanins, polyphenols, flavonoids	**Control:** Iruxol® ointment. **Authentication**: Plant material sourced from Aswan, Egypt; voucher specimen number not reported.
8	Kim et al. (2023) [22]	Human dermal fibroblasts; TGF-β1-induced skin fibrosis	Plant-cell-derived material; Gly-Pro (GP) dipeptide preparation	Gly-Pro dipeptide	**Controls**: TGF-β1-induced model and study-specific treatment controls. **Authentication:** Voucher specimen not reported. The intervention was a derived dipeptide, not a conventional whole-plant extract.
9	Kim et al. (2024) [23]	HaCaT keratinocytes and Detroit 551 fibroblasts; cytoprotection and wound healing	Callus culture; *H. sabdariffa* callus-derived polydeoxyribonucleotide (HSC-PDRN)	Polydeoxyribonucleotide	**Controls:** Sterile water and calf-thymus DNA in cell assays; epidermal growth factor (EGF) in the wound-healing assay. **Authentication:** Voucher specimen not reported.
10	Li et al. (2020) [34]	UVB-irradiated HS68 fibroblasts and B16F10 melanoma cells; photoaging, oxidative stress and hyperpigmentation	Calyx; aqueous calyx extract (HSCAE)	Phenolics, flavonoids, anthocyanins	**Controls:** Assay-specific reference compounds, including ascorbic acid, EDTA, BHA and kojic acid. **Authentication:** Refer to the botanical identification information in the cited earlier publication; include a voucher number only if documented there.
11	Malar et al. (2022) [30]	HaCaT keratinocytes exposed to phenanthrene; pollution-induced skin toxicity and premature aging	Calyx; ethanolic extract	Epigallocatechin 3-O-(3-O-methylgallate), pelargonidin 3-O-glucoside, N-feruloyltyramine, allo-aromadendrene, anisocoumarin H	**Controls:** Untreated/vehicle and phenanthrene-exposed groups. **Authentication:** Consult the cited source for botanical identification details; voucher number not established in the current article information.
12	Nassar et al. (2025) [27]	Mouse model of UV- and galactosamine-associated skin aging; photoaging and intrinsic skin aging	Calyces and epicalyces; hydroalcoholic extract formulated as Nanoliposomal gel	Chlorogenic acid derivatives, quercetin, rutin, kaempferol, myricetin, apigenin, luteolin derivatives, hibiscus acid	**Controls:** Model and treatment groups, including a green-tea-extract comparator. **Authentication:** Botanical identity reportedly authenticated by a taxonomic specialist; voucher specimen details not reported.
13	Putra et al. (2020) [32]	Primary human keloid fibroblast cultures; keloid fibrosis	Flowers; ethanolic extract	Polyphenols, anthocyanins	**Controls:** Study-specific untreated/model and treatment-comparison groups. **Authentication:** Plant identification/certification reported by the Indonesian Science Center; voucher number not reported.
14	Rambe et al. (2022) [25]	Male Wistar rats with excision wounds; wound healing and re-epithelialization	Leaves; ethanolic extract formulated as a gel	Rutin, quercetin, kaempferol, anthocyanins, chlorogenic acids	**Controls:** Gel base as negative control; Bioplacenton® as positive control. **Authentication:** Leaves collected in Tanjung Morawa, North Sumatra, Indonesia, validated by Herbarium Medanese, Universitas Sumatera Utara; voucher number not reported.
15	Shadhan and Bohari (2017) [26]	HSF 1184 human skin fibroblasts; wound-healing-related activity	Fruit; methanolic extract and solvent fractions	Not characterized	**Controls:** Specific comparator groups should be confirmed from the original article. **Authentication:** Plant occurrence documented by the Forest Research Institute Malaysia (FRIM), catalogue No. 280615-14; fruit purchased from a local herbal shop in Johor Bahru, Malaysia.
16	Wang et al. (2022) [24]	NHDF-Ad fibroblasts, HaCaT keratinocytes and RAW 264.7 macrophages; skin aging, oxidative stress and inflammation	Calyces; aqueous and ethanolic extracts, fractions, and isolated hibiscus acid	Hibiscus acid, flavonoids, polyphenols, anthocyanins	**Controls:** Assay-specific controls; quercetin (10 μM) used as a comparator in the nitric oxide assay. **Authentication:** Botanical authentication and voucher specimen not reported in the article information available. Interpret findings separately for extracts, fractions and isolated hibiscus acid.

**Table note:** Control/comparator groups are reported as described in the original studies and differed according to the experimental model and assay. Botanical authentication and voucher information are reported only when documented in the source article. Findings from isolated compounds, callus-derived preparations, and delivery-system formulations are attributed to the specific intervention evaluated rather than to crude *H. sabdariffa* L. extract generally.

**Table 3:** **Frequency and representative findings of therapeutic outcome domains reported across the 16 included preclinical studies of** ***Hibiscus sabdariffa*** **L.**

Outcome Category	Representative Outcome Measures	No. of Studies Reporting (*n* = 16)	% of Total	Summary of Reported Outcomes
Inflammatory	TNF-α, IL-6, IL-1β, NO, COX-2, iNOS	13	81.3	Reduced nitric oxide (NO) production and decreased expression of TNF-α, IL-6, IL-1β, COX-2, and iNOS in inflammatory skin models.
Oxidative Stress	ROS, MDA, SOD, CAT, GPx, TAC, GSH/GSSG	12	75	Reduced ROS and MDA levels together with increased SOD, CAT, GPx, total antioxidant capacity (TAC), and glutathione (GSH/GSSG).
Wound Healing/Tissue Regeneration	Fibroblast migration *, scratch wound closure *, wound contraction, collagen deposition, epithelial thickness, re-epithelialization, hydroxyproline	9	56.3	Increased fibroblast migration and scratch wound closure in in vitro models, and enhanced wound contraction, re-epithelialization, collagen deposition, epithelial thickness, and hydroxyproline content in in vivo models.
Anti-Melanogenic	Tyrosinase activity, melanin content, MITF	4	25	Reduced tyrosinase activity and intracellular melanin content, accompanied by downregulation of MITF expression in melanoma cell models.
Anti-Fibrotic/Photoaging	TGF-β1, MMP-1, MMP-9, TIMP-1, collagen organization, hyaluronic acid, Bcl-2, Bax	7	43.8	Reduced TGF-β1, MMP-1, and MMP-9 expression, preservation of collagen architecture, increased hyaluronic acid content, and modulation of Bcl-2/Bax expression in fibrosis and photoaging models.

Abbreviations: NO, nitric oxide; TNF-α, tumor necrosis factor-alpha; IL, interleukin; COX-2, cyclooxygenase-2; iNOS, inducible nitric oxide synthase; ROS, reactive oxygen species; MDA, malondialdehyde; SOD, superoxide dismutase; CAT, catalase; GPx, glutathione peroxidase; TAC, total antioxidant capacity; GSH/GSSG, reduced/oxidized glutathione; MITF, microphthalmia-associated transcription factor; TGF-β1, transforming growth factor-beta 1; MMP, matrix metalloproteinase; TIMP-1, tissue inhibitor of metalloproteinases-1. * Fibroblast migration and scratch wound closure were evaluated exclusively in in vitro models. Note: Frequencies and percentages represent the number and proportion of included studies reporting each outcome domain, using 16 studies as the denominator. They do not represent effect sizes, reproducibility, or therapeutic efficacy. Outcome domains were not mutually exclusive because individual studies could investigate multiple outcomes. Representative outcome measures summarize endpoints reported in the included studies and do not imply that every listed endpoint improved significantly in every study.

**Table 4:** **Molecular Mechanisms and Therapeutic Targets Reported in the Included Preclinical Studies of** ***H. sabdariffa*** **L.**

S/N	Author (Year)	Experimental Model	Major Bioactive Compound(s)	Molecular Target/Pathway	Biomarker(s) Evaluated	Reported Molecular Effect	Therapeutic Outcome	Mechanistic Evidence Level and Interpretation
1	Ticona et al. (2022) [15]	In vitro: B16F10 murine melanoma cells and RAW 264.7 macrophages	Polyphenols, flavonoids, anthocyanins, phenolic acids	Melanogenesis-associated and inflammatory responses	Tyrosinase activity, melanin content/synthesis, nitric oxide (NO)	**↓** Tyrosinase activity; ↓ melanin synthesis; ↓ NO production	Anti-melanogenic and anti-inflammatory activities	Functional evidence: Changes in tyrosinase activity, melanin synthesis, and NO support effects on pigmentation- and inflammation-associated endpoints. These findings alone do not establish a complete signaling mechanism.
2	Azizah et al. (2021) [35]	In vivo: third-degree burn model in female Wistar rats	Anthocyanins, vitamin C, citric acid, malic acid	No molecular pathway experimentally evaluated	Re-epithelialization length; inflammatory-cell infiltration	↑ Re-epithelialization; ↓ inflammatory-cell infiltration	Wound healing and skin regeneration	Tissue-level evidence: Histological changes support improved re-epithelialization and reduced inflammatory-cell infiltration. No specific molecular pathway was established.
3	Builders et al. (2013) [36]	In vivo: excision-wound model in Wistar albino rats	Flavonoids, phenolic compounds, ascorbic acid, Zn, Mn, Fe, Cu	No molecular pathway experimentally evaluated	Total flavonoid content, DPPH activity, wound closure, epithelialization time	↑ DPPH antioxidant activity; ↑ wound closure; ↓ epithelialization time	Wound healing and antioxidant activity	Functional/tissue-level evidence: Antioxidant-assay and wound-healing outcomes support biological activity, but no specific molecular pathway was experimentally evaluated.
4	Chiu et al. (2015) [37]	In vitro: human A375 melanoma cells	ECG, EGC, catechin, ellagic acid, quercetin, ferulic acid, protocatechuic acid	Intrinsic/extrinsic apoptosis; PI3K class III/Beclin1 autophagy; Akt/mTOR-associated signaling	Caspases 3/8/9, Bax, Bcl-2, t-Bid, cytochrome c, Fas/FasL, PI3K class III, Beclin1, ATG5/9/16, LC3-II, p62, Akt/p-Akt, mTOR/p-mTOR	↑ caspases 3/8/9, Bax, t-Bid, cytochrome c, Fas/FasL, PI3K class III, Beclin1, ATG5/9/16, LC3-II; ↓ Bcl-2, p-Akt, p-mTOR	Apoptosis/autophagy induction and inhibition of melanoma-cell proliferation	Molecular/functional evidence: Changes in apoptosis-, autophagy-, and Akt/mTOR-associated markers support these cellular responses in the tested melanoma model. The findings should not be generalized to other pathways without direct measurements.
5	Cho et al. (2023) [17]	In vitro: human HaCaT keratinocytes and murine B16F1 melanoma cells	Water-soluble phytochemicals, flavonoids, anthocyanins	Skin-barrier regulation; antioxidant defense; melanogenesis-associated responses; transcriptomic regulation	FLG, SOD1, melanin content, BNIP3, RNASE7, MIR210HG, SAMD4A, PPP1R3C, SLC6A8, AKR1B10, ALDH1A1, NME1, EGLN1/3, LOXL2, ADM, STC2, NDRG1, HK2, PDK1, ITGA5	↑ FLG; ↑ SOD1; ↓ melanin content; differential regulation of transcriptomic markers	Skin-barrier function, antioxidant activity, anti-melanogenic effects, and skin homeostasis	Transcriptomic and functional evidence: Differential gene expression, including FLG and SOD1, alongside changes in melanin content, supports barrier-, antioxidant-, and pigmentation-associated effects. Transcript changes do not establish corresponding protein activation.
6	Hwang et al. (2013) [33]	In vitro: α-MSH-stimulated human A375 melanoma cells	Liposome-encapsulated anthocyanins	MITF–tyrosinase-associated melanogenesis	Tyrosinase activity and protein, MITF protein, melanin content	↑ antioxidant activity; ↓ melanin synthesis; ↓ tyrosinase activity and protein; ↓ MITF protein	Suppression of melanogenesis and photoprotective activity	Molecular and functional evidence: Changes in MITF and tyrosinase protein, tyrosinase activity, and melanin content support a melanogenesis-associated mechanism in this specific cell model and preparation.
7	Khalil et al. (2022) [31]	In vivo: superficial burn model	Anthocyanin-rich ethanolic flower extract	Inflammatory signaling; TGF-β-associated wound remodeling	TLR4, TNF-α, IL-6, TGF-β, total antioxidant capacity (TAC), collagen deposition	↓ TLR4; ↓ TNF-α; ↓ IL-6; ↓ TGF-β; ↑ TAC; ↓ collagen deposition	Burn-wound healing, epithelialization, and tissue regeneration	Inflammatory and tissue-level evidence: Changes in TLR4, cytokines, TGF-β, TAC, and collagen deposition support altered inflammatory and remodeling responses. Direct NF-κB inhibition should not be claimed unless pathway-specific endpoints were measured.
8	Kim et al. (2023) [22]	In vitro: TGF-β1-stimulated human dermal fibroblasts	Gly-Pro dipeptide	TGF-β1/Smad2/3–ATF4–serine/glycine biosynthesis-associated pathway	p-Smad2/3, ATF4, COL1A1, COL1A2, COL3A1, procollagen I, PHGDH, PSAT1, PSPH, SHMT2, SLC2A1	↓ p-Smad2/3; ↓ ATF4; ↓ COL1A1/COL1A2/COL3A1; ↓ procollagen I and listed biosynthesis-associated markers	Antifibrotic activity and reduced collagen biosynthesis	Direct pathway-associated evidence: Reduced phosphorylated Smad2/3 and collagen-related markers support modulation of TGF-β1/Smad-associated responses by the tested Gly-Pro preparation. Do not generalize this finding to all *H. sabdariffa* extracts.
9	Kim et al. (2024) [23]	In vitro: human HaCaT keratinocytes and Detroit 551 fibroblasts	Polydeoxyribonucleotide (PDRN) derived from *H. sabdariffa* callus	Nrf2-associated antioxidant signaling	Nrf2, MMP2, MMP9, KGF, VEGF, SOD2, CAT, AQP3, TGF-β1, FGF21, PIP	↑ Nrf2 transcript; ↑ MMP9, KGF, VEGF, SOD2, CAT, AQP3 and PIP; ↓ MMP2 (as reported in the table)	Keratinocyte proliferation, collagen synthesis, antioxidant defense, and skin regeneration	Transcript-level and protein-level evidence: Increased Nrf2 transcript and reported changes in antioxidant/repair-associated proteins support an Nrf2-associated response. Do not claim Nrf2 nuclear translocation or direct activation unless specifically measured.
10	Li et al. (2020) [34]	In vitro: UVB-exposed HS68 fibroblasts and B16F10 melanoma cells	Anthocyanins, flavonoids, gallic acid, caffeic acid, syringic acid	Oxidative stress; MMP/TIMP balance; MITF–tyrosinase/TRP-associated pigmentation	ROS, MMP-1, MMP-3, TIMP-1, COL1A1/collagen, MITF, tyrosinase, TRP-1, TRP-2	↓ ROS; ↓ MMP-1/MMP-3; ↑ TIMP-1 and collagen; ↓ MITF, tyrosinase, TRP-1, TRP-2	Anti-photoaging, antioxidant, and anti-melanogenic activities	Molecular and functional evidence: Changes in MITF, tyrosinase, TRP-1/TRP-2, ROS, MMPs, TIMP-1, and collagen support pigmentation- and photoaging-associated effects in the tested models.
11	Malar et al. (2022) [30]	In vitro: phenanthrene-exposed HaCaT keratinocytes	Ethanolic calyx extract	CAR/PXR/RXR–CYP-associated xenobiotic metabolism; oxidative-stress response	ROS, mitochondrial membrane potential (ΔΨm), CYP1A1, CYP1B1, CYP2B, CYP3A4, CAR, PXR, RXR, NRF2, NQO1, Bcl-2, Bax	↓ CAR/PXR/RXR and CYP-associated markers (as reported); ↓ ROS; ↑ ΔΨm; ↑ NRF2; ↑ NQO1; ↑ Bcl-2; ↓ Bax	Protection against pollutant-induced oxidative skin injury	Pathway-associated and functional evidence: Changes in ROS, NRF2, NQO1, and other measured endpoints support an antioxidant/xenobiotic-response association. Specify NRF2 measurement level only after checking the original article; these findings alone do not prove pathway causality.
12	Nassar et al. (2025) [27]	In vivo: UV- and galactosamine-induced skin-aging model	Phenolic acids, anthocyanins, flavonoids in nanoliposomes	Oxidative stress; MMP/TIMP balance; apoptosis; extracellular-matrix remodeling	GSH, MDA, MMP-1/2/9, TIMP-1, collagen IV, Bcl-2, Bax	↑ GSH; ↓ MDA; ↓ MMP-1/2/9; ↑ TIMP-1 and collagen IV; ↑ Bcl-2; ↓ Bax	Reduced photoaging, preserved collagen integrity, and dermal repair	Biochemical and tissue-associated evidence: Changes in redox, matrix-remodeling, and apoptosis-associated markers support protective effects in this animal model. The network should not imply direct pathway activation unless directly assessed.
13	Putra et al. (2020) [32]	In vitro: human keloid fibroblasts	Anthocyanin-rich polyphenol extract	TGF-β1-associated fibrotic response	TGF-β1, fibroblast apoptosis, fibroblast viability	↓ TGF-β1; ↑ fibroblast apoptosis	Antifibrotic activity and suppression of fibroblast proliferation	Molecular and cellular evidence: Reduced TGF-β1 and increased fibroblast apoptosis support an antifibrotic interpretation in this keloid-fibroblast model. Direct Smad inhibition should not be inferred without pathway-specific measurements.
14	Shadhan and Bohari (2017) [26]	In vitro: HSF1184 fibroblast scratch assay	Methanolic fruit extract and solvent fractions	Fibroblast migration/proliferation; α-glucosidase inhibition	α-Glucosidase activity, glucose diffusion, cell viability, scratch-wound closure	↑ α-glucosidase inhibition; ↓ glucose diffusion; ↑ fibroblast viability, migration, and wound closure	In vitro wound-healing-associated activity; antidiabetic activity reported	Functional evidence: Fibroblast viability, migration, and scratch closure support a wound-healing-associated response in vitro. These outcomes do not establish a specific molecular signaling pathway.
15	Rambe et al. (2022) [25]	In vivo: excision-wound model in male Wistar rats	Roselle leaf extract (quercetin, rutin, kaempferol, chlorogenic acids, anthocyanins)	Inflammatory signaling; oxidative stress; tissue repair	Wound contraction, wound-healing percentage, epithelial thickness	↑ wound contraction; ↑ wound healing; ↑ epithelial thickness and re-epithelialization	Accelerated wound healing and epithelial regeneration	Tissue-level/functional evidence: Wound contraction and epithelialization outcomes support improved repair in this animal model. Specific inflammatory or oxidative pathways should be described as hypothesized unless directly measured.
16	Wang et al. (2022) [24]	In vitro: NHDF-Ad fibroblasts, HaCaT keratinocytes, and RAW264.7 macrophages	Hibiscus acid	Oxidative-stress response; extracellular-matrix remodeling; inflammatory-associated responses	Collagen, hyaluronic acid, NO, cell viability, extracellular ATP, protein carbonyls, GSH/GSSG ratio	↑ collagen and hyaluronic acid; ↓ NO, extracellular ATP, and protein carbonyls; ↑ cell viability and GSH/GSSG ratio	Antioxidant, anti-inflammatory-associated, anti-aging, and skin-protective activities	Functional/biochemical evidence: Changes in NO, extracellular ATP, protein carbonyls, GSH/GSSG, viability, collagen, and hyaluronic acid support redox-, inflammatory-, and matrix-associated effects. These endpoints alone do not demonstrate direct NF-κB or MAPK inhibition.

Note: ↑, increased; ↓, decreased. Arrow directions indicate the reported changes in the measured outcomes in the original studies.

### 3.8. Risk of Bias Assessment and Interpretation of Evidence Certainty

**Figure 4:**
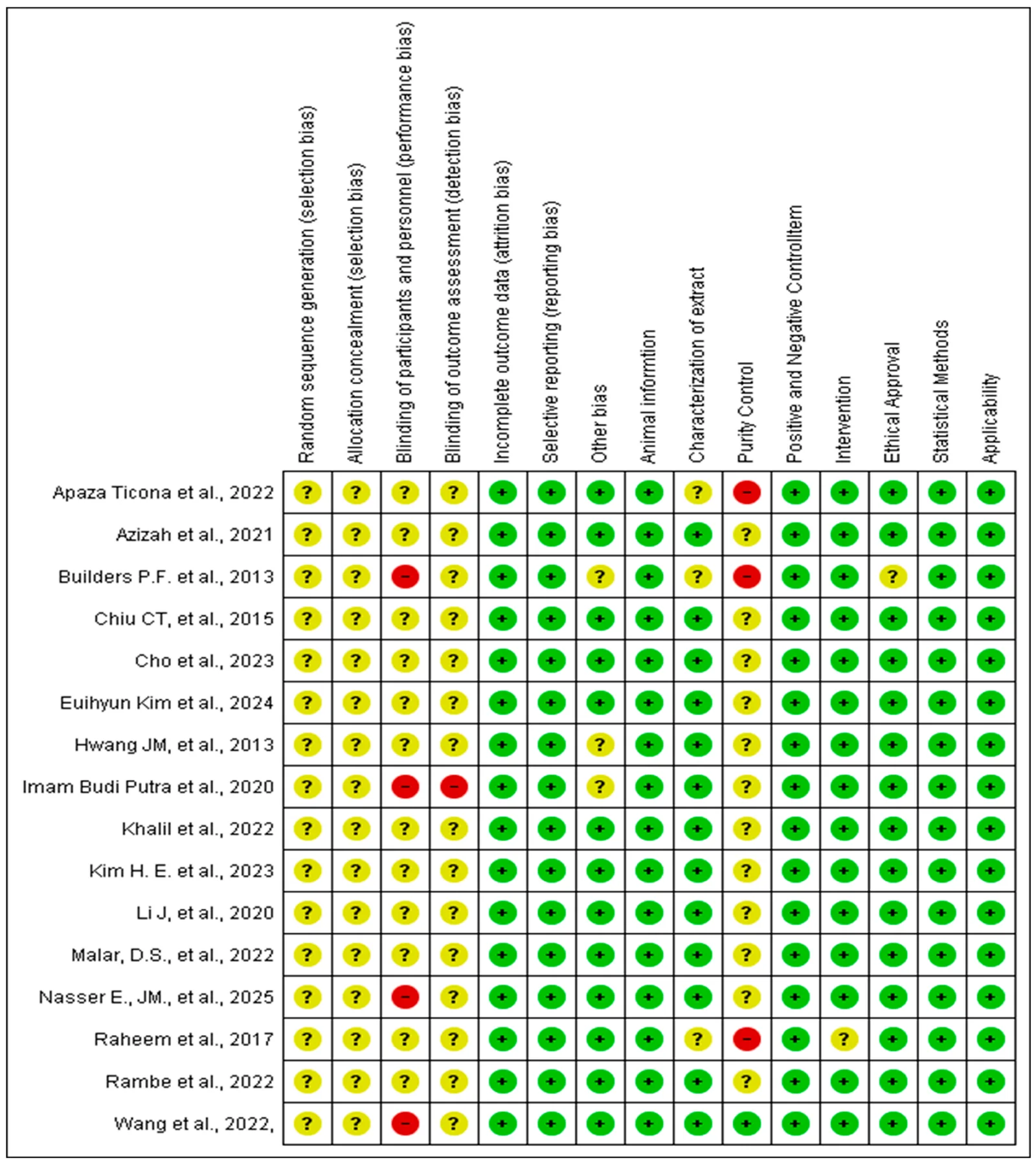
Study-level risk of bias assessment of the included preclinical studies using the SYRCLE Risk of Bias tool. Each row represents an individual study, and each column represents an assessed methodological domain. Green circles (+) indicate low risk of bias, yellow circles (?) indicate unclear risk of bias, and red circles (−) indicate high risk of bias. Source: Prepared by the authors [15,17,24,25,26,27,28,29,30,31,32,33,34,35,36,37].

**Figure 5:**
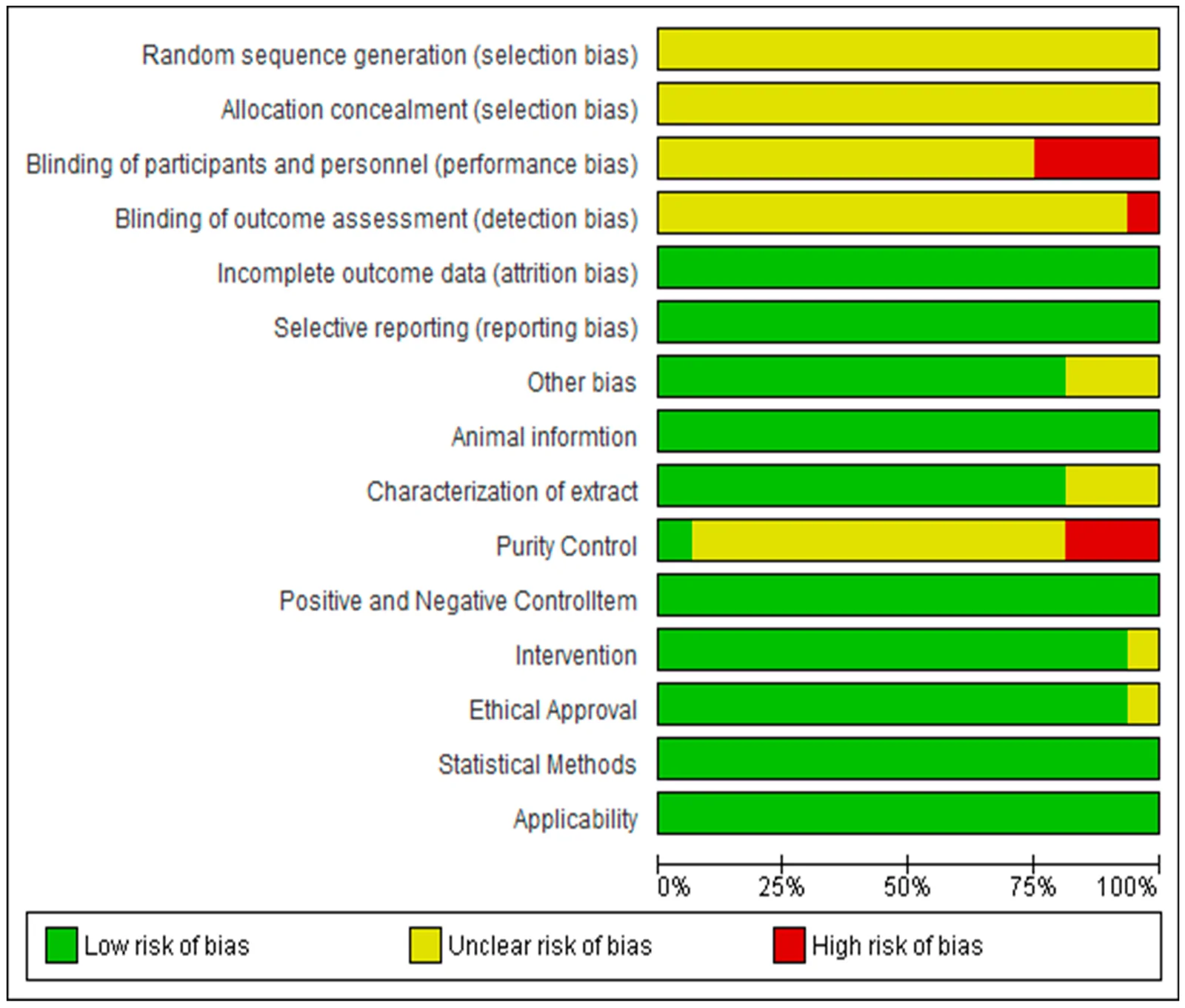
Overall risk of bias summary of the included preclinical studies based on the SYRCLE Risk of Bias tool. The stacked bar chart presents the percentage of studies classified as having low risk of bias (green), unclear risk of bias (yellow), and high risk of bias (red) across each methodological domain. Source: Prepared by the authors.

Figure 4 and Figure 5 summarize the risk-of-bias assessments conducted using the SYRCLE Risk of Bias tool, supplemented by criteria relevant to preclinical phytopharmacological research. The study-level assessment identified methodological reporting limitations across the 16 included studies. Random sequence generation and allocation concealment were rated as unclear in all 16 studies because the available assessments did not establish whether these procedures were adequately implemented. The ratings for blinding of participants/personnel and blinding of outcome assessment require consistency with the final study-level assessment table and are therefore not quantified here. All studies were rated as having low risk for incomplete outcome data, selective reporting, animal information, positive and negative controls, statistical methods, and applicability. Extract characterization was rated as low risk in 13 studies and unclear in three, with no high-risk ratings. Purity control was rated as low risk in one study, unclear in 12, and high risk in three. Intervention procedures and ethical approval were predominantly rated as low risk, with isolated unclear assessments.

These findings indicate that, although several methodological and reporting domains were rated as low risk, uncertainty remains in important areas. In particular, insufficient information on randomization and allocation concealment limits confidence in the internal validity of the experiments. Uncertainty regarding blinding may also affect the interpretation of outcomes, depending on the experimental design and the objectivity of the measurements. Variability in extract characterization and purity control is relevant because differences in the composition and quality of *Hibiscus sabdariffa* L. preparations may influence their biological activity and reproducibility. These limitations should be considered alongside the heterogeneity in plant materials, extraction procedures, formulations, concentrations, experimental conditions, and outcome measures across the included studies.

The frequency of reported outcomes provides a descriptive overview of the research landscape but should not be equated with the strength or certainty of the evidence. Anti-inflammatory effects were reported in 13 of the 16 studies (81.3%), antioxidant effects in 12 (75.0%), and wound-healing or tissue-regenerative effects in nine (56.3%). These proportions describe how frequently the respective outcomes were reported; they do not quantify effect magnitude, reproducibility, consistency across independent models, or causal certainty. Interpretation should also consider the direction and consistency of findings, the relevance and diversity of experimental models, the adequacy of controls, methodological limitations, and whether proposed mechanisms were directly measured. Accordingly, the findings identify recurring biological patterns but do not independently establish definitive causal mechanisms or therapeutic efficacy.

Publication bias represents an additional potential source of uncertainty. Because this review synthesized published studies, statistically significant, positive, or biologically favorable findings may be overrepresented relative to null, inconsistent, or unfavorable results. This possibility is relevant to exploratory preclinical research, in which small experiments, variable outcome measures, and differences in experimental design may limit reproducibility. However, the presence, direction, and magnitude of publication bias could not be established from the available evidence. Funnel plots and statistical tests for small-study effects were not performed because the studies were heterogeneous and no quantitative meta-analysis was conducted. Furthermore, low-risk ratings for selective reporting within individual studies do not exclude publication bias across the literature as a whole. The possibility of overrepresentation of favorable findings should therefore be acknowledged without assuming that publication bias was definitively present.

A formal certainty-of-evidence framework, such as GRADE, was not applied because the evidence comprised heterogeneous preclinical in vitro and in vivo investigations that differed in experimental models, interventions, and outcome measurements, precluding quantitative synthesis. Instead, the evidence was interpreted qualitatively, considering the number of supporting studies, the consistency and direction of findings, the diversity and relevance of models, methodological limitations, and the directness of the measured outcomes. No formal numerical certainty ratings were assigned. This approach provides a more cautious interpretation than study counts alone while recognizing that the strength and reproducibility of the reported effects remain incompletely established.

Overall, the risk-of-bias assessment and qualitative interpretation of evidence indicate that *H. sabdariffa* L. has promising preclinical dermatological potential, particularly in relation to inflammation, oxidative stress, and tissue repair. However, methodological uncertainties, variation in intervention characterization, heterogeneity across experimental systems, and the possibility of publication bias limit confidence in the consistency, reproducibility, and translational relevance of these findings. Future studies should provide transparent descriptions of randomization, allocation concealment, and blinding; standardized phytochemical characterization and appropriate quality-control procedures; adequately powered and independently replicated experiments; and complete reporting of positive, null, and unfavorable results. These improvements would strengthen the reliability of the evidence and support a more robust assessment of the therapeutic potential of *H. sabdariffa* L. (Figure 4 and Figure 5).

### 3.9. Limitations of the Review Process

This review has several methodological limitations. First, the search was restricted to four bibliographic databases and published literature, potentially excluding relevant studies indexed elsewhere or unpublished findings. Second, although two reviewers independently conducted study selection, data extraction, and risk-of-bias assessment, some subjectivity in methodological judgments may remain. Third, the substantial heterogeneity among the included studies in terms of experimental models, extract preparations, interventions, outcome measures, and study designs precluded quantitative meta-analysis. Consequently, formal statistical assessment of reporting bias and quantitative synthesis of the certainty of evidence were not performed. Finally, the possibility of publication bias and selective reporting cannot be ruled out, and the findings should therefore be interpreted cautiously, particularly when considering their translation to human dermatological applications.

## 4. Conclusions

This systematic review identified promising but preliminary preclinical evidence supporting the dermatological potential of *Hibiscus sabdariffa* L. Across the 16 included studies, reported effects encompassed anti-inflammatory, antioxidant, wound-healing, anti-melanogenic, anti-photoaging, and anti-fibrotic activities. These findings suggest that *H. sabdariffa* L. and its derived compounds may influence biological processes associated with oxidative stress, inflammatory signaling, melanogenesis, extracellular matrix remodeling, and skin repair. However, the evidence remains exclusively preclinical, and the substantial heterogeneity in plant materials, extraction methods, formulations, experimental models, and methodological reporting limits comparisons across studies and the assessment of the consistency and strength of the findings. Therefore, the current evidence supports further investigation rather than definitive conclusions about therapeutic efficacy in humans. In addition, because the literature search was not updated after 24 March 2025, relevant subsequent studies may have been missed. Future research should prioritize standardized phytochemical characterization, rigorous and reproducible experimental designs, stronger mechanistic validation, and well-designed translational and clinical studies to establish safety, dose–response relationships, optimal formulations, and clinical effectiveness.

## Data Availability

No new data were generated for this systematic review. The evidence synthesized in this study was extracted from previously published articles cited in the manuscript. The data extraction form, study-level extracted data, and other materials used in the review are available from the corresponding author upon reasonable request. No analytic code was used for quantitative meta-analysis because no meta-analysis was performed.

## Supplementary Materials

The following supporting information can be downloaded at: https://www.mdpi.com/article/10.3390/ijms27219473/s1.
