# Supplementary material for: Dermatoprotective Potential of Hibiscus sabdariffa L.: A Systematic Review of Preclinical Evidence
Source: Int J Mol Sci. 2026 Oct 23;27(21):9473. doi: 10.3390/ijms27219473 (PMC13762903; doi:10.3390/ijms27219473)
Supplement: Supplementary file 1 [file ijms-27-09473-s001.zip › ijms-4606622-supplementary.pdf]

Table S1. Complete Database-Specific Search Strategies Used for the Systematic Review

Data base	Descriptors	Items Found	Time	Date
P<br>U<br>B<br>M<br>E<br>D	#**Filter 1: *Hibiscus sabdariffa***<br>"hibiscus"[MeSH Terms] OR *Hibiscus sabdariffa*[TIAB] OR "hibiscus"[MeSH Terms] OR Roselle[TIAB] OR "hibiscus"[MeSH Terms] OR Roselles[TIAB] OR "Malvaceae"[MeSH Terms] OR Malvaceae[TIAB] OR "hydroxycitric acid"[TIAB] OR hydroxycitric acid[TIAB] OR "hydroxycitric acid"[TIAB] OR hibiscus acid[TIAB]	16,996	2:20 PM	March 24, 2025
	#**Filter 2: Skin diseases**<br>"skin diseases"[MeSH Terms] OR Skin diseases[TIAB] OR "dermatitis"[MeSH Terms] OR Dermatitis[TIAB] OR "skin diseases"[MeSH Terms] OR Dermatoses[TIAB] OR "skin diseases"[MeSH Terms] OR Skin and Subcutaneous Tissue Disorders[TIAB] OR "psoriasis"[MeSH Terms] OR Psoriasis[TIAB] OR "eczema"[MeSH Terms] OR Eczema[TIAB] OR ("skin"[MeSH Terms] OR Skin[TIAB]) OR ("regeneration"[MeSH Terms] OR Regeneration[TIAB]) OR "wound healing"[MeSH Terms] OR Wound Healing[TIAB] OR "skin neoplasms"[MeSH Terms] OR Skin cancer[TIAB]	2,217,455	2:21 PM	March 24, 2025
	# **Filter 3: Therapeutic**<br>"anti-inflammatory agents"[TIAB] OR "anti-inflammatory agents"[MeSH Terms] OR Anti-Inflammatory Agents[TIAB] OR "antioxidants"[TIAB] OR "antioxidants"[MeSH Terms] OR Antioxidants[TIAB] OR "wound healing"[MeSH Terms] OR wound healing[TIAB]	616,855	2:22 PM	March 24, 2025
	#**1 AND** #**2 AND** #**3**	158	2:22 PM	March 24, 2025
**Data base**	**Descriptors**	**Items Found**	**Time**	**Date**
S<br>C<br>O<br>P<br>U<br>S	#**Filter 1: *Hibiscus sabdariffa***<br>TITLE-ABS-KEY ("hibiscus") OR TITLE-ABS-KEY ("Hibiscus sabdariffa") OR TITLE-ABS-KEY (" Roselle ") OR TITLE-ABS-KEY ("Roselles") OR TITLE-ABS-KEY ("Malvaceae") OR TITLE-ABS-KEY ("hydroxycitric acid") OR TITLE-ABS-KEY ("hibiscus acid")	15,381	2:25 PM	March 24, 2025
	#**Filter 2: Skin diseases**<br>*TITLE-ABS-KEY ("dermatitis") OR TITLE-ABS-KEY ("skin diseases") OR TITLE-ABS-KEY ("Dermatoses") OR TITLE-ABS-KEY ("Skin and Subcutaneous Tissue Disorders") OR TITLE-ABS-KEY ("psoriasis") OR TITLE-ABS-KEY ("eczema") OR TITLE-ABS-KEY ("skin regeneration") OR TITLE-ABS-KEY ("wound healing") OR TITLE-ABS-KEY ("skin neoplasms") OR TITLE-ABS-KEY ("skin cancer")*	831,378	2:26 PM	March 24, 2025

	# **Filter 3: Therapeutic**<br>TITLE-ABS-KEY ("anti-inflammatory agents") OR TITLE-ABS-KEY ("antioxidants") OR TITLE-ABS-KEY ("wound healing")	1,036,609	2:27 PM	March 24, 2025
	**#1 AND #2 AND #3**	185	2:28 PM	March 24, 2025
**Data base**	**Descriptors**	**Items Found**	**Time**	**Date**
**WEB OF SCIENCE**	**#Filter 1: *Hibiscus sabdariffa***<br>TS=(hibiscus) OR TS=(*Hibiscus sabdariffa*) OR TS= (Roselle) OR TS=(Roselles) OR TS= (Malvaceae) OR TS=(hydroxycitric acid) OR TS=(Hibiscus acid)	10,300	2:31 PM	March 24, 2025
	**#Filter 2: Skin diseases**<br>TS=(dermatitis) OR TS=(skin diseases) OR TS=(Dermatoses) OR TS=(Skin and Subcutaneous Tissue Disorders) OR TS=(psoriasis) OR TS=(eczema) OR TS=(skin regeneration) OR TS=(wound healing) OR TS=(skin neoplasms) OR TS=(skin cancer)	575,800	2:32 PM	March 24, 2025
	# **Filter 3: Therapeutic**<br>TS=(anti-inflammatory agents) OR TS=(antioxidants) OR TS=(wound healing)	775,922	2:33 PM	March 24, 2025
	**#1 AND #2 AND #3**	81	2:34 PM	March 24, 2025
**Database**	**Descriptors**	**Items Found**	**Time**	**Date**
**EMBASE**	**#Filter 1: *Hibiscus sabdariffa***<br>'hibiscus'/exp OR hibiscus OR 'hibiscus sabdariffa'/exp OR 'hibiscus sabdariffa' OR 'roselle'/exp OR roselle OR 'hibiscus acid'/exp OR 'hibiscus acid' OR 'roselle'/exp OR roselle OR 'roselles'/exp OR roselles OR 'Malvaceae'/exp OR Malvaceae OR 'hydroxycitric acid'/exp OR hydroxycitric acid	7,159	2:42 PM	March 24, 2025
	**#Filter 2: Skin diseases**<br>'dermatitis'/exp OR dermatitis OR 'skin diseases'/exp OR skin diseases/exp OR 'dermatoses'/exp OR dermatoses OR 'Skin and Subcutaneous Tissue Disorders'/exp OR Skin and Subcutaneous Tissue Disorders OR 'eczema'/exp OR eczema OR 'rosacea'/exp OR rosacea OR ‘psoriasis’/exp OR psoriasis OR ‘skin regeneration’/exp OR skin regeneration OR ‘wound healing’/exp OR wound healing OR ‘skin neoplasms’/exp OR skin neoplasms OR ‘skin cancer’/exp OR skin cancer	309,375	2:43 PM	March 24, 2025

	# **Filter 3: Therapeutic**<br>'anti-inflammatory agents'/exp OR anti-inflammatory OR 'antioxidant'/exp OR antioxidant OR 'wound healing'/exp OR wound healing	281,416	2:43 PM	March 24, 2025
	**#1 AND #2 AND #3**	113	2:52 PM	March 24, 2025

Supplementary Table S2. Study-level risk-of-bias and methodological quality assessment

Part A. Study-level risk-of-bias ratings. Risk-of-bias judgments are presented using a traffic-light classification: low risk (L, green), unclear risk (U, yellow), and high risk (H, red). Study names and publication years are presented as reported in the original articles.

Study	Random sequence generation	Allocation concealment	Blinding of participants/personnel	Blinding of outcome assessment	Incomplete outcome data	Selective reporting	Other bias	Animal information	Characterization of extract	Purity control	Positive/negative control item	Intervention	Ethical approval	Statistical methods	Applicability
Ticona et al., 2022 [15]	U	U	U	U	L	L	L	L	U	H	L	L	L	L	L
Azizah et al., 2020 [35]	U	U	U	U	L	L	L	L	L	U	L	L	L	L	L
Builders P.F. et al., 2013 [36]	U	U	H	U	L	L	U	L	U	H	L	L	U	L	L
Chiu C.T. et al., 2015 [37]	U	U	U	U	L	L	L	L	L	U	L	L	L	L	L

Cho et al., 2023 [17]	U	U	U	U	L	L	L	L	L	U	L	L	L	L	L
Kim et al., 2024 [23]	U	U	U	U	L	L	L	L	L	U	L	L	L	L	L
Hwang J.M. et al., 2013 [33]	U	U	U	U	L	L	U	L	L	U	L	L	L	L	L
Putra et al., 2020 [32]	U	U	H	H	L	L	U	L	L	U	L	L	L	L	L
Khalil et al., 2022 [31]	U	U	U	U	L	L	L	L	L	U	L	L	L	L	L
Kim H.E. et al., 2023 [22]	U	U	U	U	L	L	L	L	L	U	L	L	L	L	L
Li J. et al., 2020 [34]	U	U	U	U	L	L	L	L	L	U	L	L	L	L	L
Malar D.S. et al., 2022 [30]	U	U	U	U	L	L	L	L	L	U	L	L	L	L	L
Nasser E.J.M. et al., 2025 [27]	U	U	H	U	L	L	L	L	L	U	L	L	L	L	L

Shadhan, R.M.; Bohari et al., 2017 [26]	U	U	U	U	L	L	L	L	U	H	L	U	L	L	L
Rambe et al., 2022 [25]	U	U	U	U	L	L	L	L	L	U	L	L	L	L	L
Wang et al., 2022 [24]	U	U	H	U	L	L	L	L	L	L	L	L	L	L	L

Part B. Risk-of-Bias Assessment and Interpretation of Ratings

Risk-of-bias judgments are presented at the domain level using a traffic-light classification: low risk (L), unclear risk (U), and high risk (H). No overall numerical quality score was calculated because the assessment focused on domain-specific judgments rather than a composite scoring system. An unclear rating indicates that the available information was insufficient to determine the risk of bias and should not be interpreted as evidence of low risk. The SYRCLE risk-of-bias tool was applied to animal intervention studies, while supplementary methodological criteria were considered for in vitro studies, as appropriate. Judgments were based on the methods and results reported in the original articles and the corresponding assessment records.

Note: L = low risk; U = unclear risk; H = high risk. Ratings are domain-specific and should not be interpreted as an overall numerical quality score.

**Domain**	**Summary of ratings**	**Interpretation**
Random sequence generation	Unclear risk in all 16 studies	Random sequence generation was insufficiently reported to establish whether allocation sequences were generated appropriately.
Allocation concealment	Unclear risk in all 16 studies	Reporting was insufficient to determine whether allocation was concealed before assignment to study groups.
Blinding of participants/personnel	High risk: Builders et al. (2013) [36], Putra et al. (2020) [32], Nasser et al. (2025) [27], and Wang et al. (2022) [24]. Unclear risk in the remaining studies.	The ratings reflect the judgments recorded in the risk-of-bias assessment.
Blinding of outcome assessment	High risk: Putra et al. (2020) [32]. Unclear risk in the remaining studies.	The ratings reflect the judgments recorded in the risk-of-bias assessment.

Other bias	Unclear risk: Builders et al. (2013) [32], Hwang et al. (2013) [33], and Putra et al. (2020) [32].	The available assessment records classified these studies as having unclear risk in this domain.
Characterization of extract	Unclear risk: Ticona et al. (2022) [15], Builders et al. (2013) [36], and Shadhan, R.M.; Bohari, et al. (2017) [26]. Low risk in the remaining studies.	The ratings reflect the recorded assessment of botanical identity, plant material, extraction procedures, and phytochemical characterization.
Purity control	High risk: Ticona et al. (2022) [15], Builders et al. (2013) [36], and Shadhan, R.M.; Bohari, et al. (2017) [26]. low risk: Wang et al. (2022) [24]; unclear risk in the remaining 12 studies.	The ratings reflect the recorded assessment of purity and relevant quality-control information.
Intervention	Unclear risk: Shadhan, R.M.; Bohari, et al. (2017) [26]. Low risk in the remaining studies.	The ratings reflect the recorded assessment of intervention description and delivery.
Ethical approval	Unclear risk: Builders et al. (2013) [36]. Low risk in the remaining studies.	The rating indicates uncertainty based on the information recorded in the assessment; it does not establish that ethical approval was absent.

Supplementary Table S3. Study-level characteristics of *Hibiscus sabdariffa* L. preparations evaluated in preclinical skin-related models.

Study	Plant part/ material	Extraction solvent/method	Preparation/ formulation	Dose/ concentration	Route/model	Treatment/ exposure duration	Phytochemical characterization	Controls/ comparators
Ticona et al., 2022 [15]	Leaves	Distilled-water extraction of dried leaf powder (1 kg/2 L); freeze-dried. A polyphenol-rich fraction was prepared using acidified ethanol for phenolic analysis.	Aqueous extract (AEHS); polyphenol-rich fraction used for chemical characterization	0.20–100 μg/mL screening range; individual assays used selected concentrations	In vitro: B16F10 murine melanoma cells and RAW 264.7 macrophages	72 h for tyrosinase/melanin assays; NO assay: extract pretreatment followed by LPS stimulation (see article protocol)	Total polyphenols 15.97 ± 0.49 mg GAE/g; 1H-NMR identified phenolic acids, flavonoids and anthocyanins	Kojic acid (melanogenesis); L-NMMA (NO assay); actinomycin D (cytotoxicity)
Azizah et al., 2020 [35]	Calyces	Methanolic/ethanolic extraction as described in the article	Extract evaluated in a rat burn model by oral and topical administration	250 mg/kg reported; topical application amount should	In vivo: female Wistar rats with	16 days of treatment; assessment	Anthocyanins, vitamin C, citric acid, malic acid and	Cold-cream base; silver sulfadiazine

				be transcribed from the original protocol if separately specified	third-degree burns	reported on day 17	proteins as reported in the study	
Builders P.F. et al., 2013 [36]	Calyces	Methanolic extraction	Creams containing *H. sabdariffa* extract; extract/gentamicin combination groups also included	1%, 5% and 10% extract creams; additional gentamicin/combination arms per article	In vivo: rat excision-wound model; topical treatment	21 days	Flavonoids, phenolic compounds, ascorbic acid and trace minerals reported	Placebo cream; gentamicin cream
Chiu C.T. et al., 2015 [37]	Leaves	Polyphenolic leaf extract (HLP); extraction/fractionation procedures reported in the article	Polyphenol-rich leaf extract	Assay-specific concentrations; do not infer a single dose across endpoints	In vitro: melanoma-cell melanogenesis models and skin-related cells	Assay-specific; 24 h reported for selected cell/viability procedures	Individual phenolic compounds reported, including epicatechin gallate, catechin, ellagic acid and protocatechuic acid	Assay-specific controls as reported in the article
Cho et al., 2023 [17]	Leaves and callus cells	Hot-water extraction	*H. sabdariffa* plant extract (HSPE) and callus-cell extract (HSCE)	Concentration varied by assay; use assay-specific values from original article	In vitro: HaCaT keratinocytes and B16F1 melanoma cells	Assay-specific	Reported constituents included guanine, phenylalanine, tryptophan, adenine, uridine, adenosine and guanosine	Water/vehicle and assay-specific positive controls (e.g., kojic acid or NAC where applicable)
Kim et al., 2024 [23]	Calyx-derived anthocyanins	Anthocyanin preparation; liposomal encapsulation	Free anthocyanin and anthocyanin-loaded liposomes	Concentrations are assay-specific; verify units directly against the article before	In vitro: melanoma-cell model	Assay-specific	Anthocyanins; encapsulation/formulation compared with free preparation	Free anthocyanin and formulation/vehicle controls as reported

				final journal submission				
Hwang J.M. et al., 2013 [33]	Flowers	372.58 g flower powder suspended in 1,000 mL of 70% ethanol for 48 h at room temperature; filtered and concentrated by rotary evaporation	10% (w/w) extract ointment in simple ointment base (liquid paraffin and white soft paraffin)	10% (w/w) ointment	In vivo: male Wistar rats with second-degree contact burns; topical application	Applied three times daily; skin tissue collected after 36 h	Qualitative screening reported phenolic compounds and flavonoids among other phytochemical groups; do not imply quantitative anthocyanin profiling unless separately supported	Ointment base/placebo; Iruxol® ointment
Putra et al., 2020 [32]	Plant-cell-derived material associated with H. sabdariffa	Not a conventional solvent extract; Gly-Pro dipeptide preparation derived from plant-cell material	Gly-Pro dipeptide	Assay-specific; report only the values stated in the original article	In vitro: human dermal fibroblasts in TGF-β1-induced fibrotic/collagen-response model	Assay-specific	Gly-Pro (glycine–proline) dipeptide; specific preparation rather than crude-extract phytochemistry	Untreated/model controls and assay-specific controls as reported
Khalil et al., 2022 [31]	H. sabdariffa callus culture	Callus-derived polydeoxyribonucleotide preparation	HSC-PDRN	1, 2, 5 and 10 μg/mL in cell experiments	In vitro: HaCaT keratinocytes and Detroit 551 fibroblasts; wound-related cellular assays	24–48 h for cell-treatment experiments; assay-specific wound-assay schedule	Polydeoxyribonucleotide (PDRN); not a conventional plant extract	Distilled water (negative control); calf-thymus DNA (positive control); EGF in wound-related assay where specified

Kim H.E. et al., 2023 [22]	Calyces	Aqueous extraction; cold-water extraction at 4 °C for 24 h, followed by concentration and lyophilization	Aqueous calyx extract (HSCAE)	Assay-specific concentrations; report separately for UVB/photoaging and melanogenesis endpoints	In vitro: UVB-exposed skin fibroblasts and B16F10 melanoma cells	Assay-specific; 24 h reported for selected cell experiments	Phenolics, flavonoids and anthocyanins; phenolic acids reported in the article	Endpoint-specific controls, including ascorbic acid/EDTA/BHA or kojic acid where applicable
Li J. et al., 2020 [34]	Calyces	Ethanolic extraction	Ethanolic calyx extract	Protective-effect experiments used extract pretreatment; phenanthrene challenge 250 µM; extract concentrations varied by assay	In vitro: HaCaT keratinocytes exposed to phenanthrene	6 h extract pretreatment before phenanthrene exposure	Reported compounds included epigallocatechin 3-O-(3-O-methylgallate), pelargonidin 3-O-glucoside, allo-aromadendrene, anisocoumarin H and N-feruloyltyramine	Untreated cells and phenanthrene-exposed model control
Malar D.S. et al., 2022 [30]	Calyces and epicalyces	Hydroalcoholic extract; preparation and nanoliposome formulation described in the article	H. sabdariffa extract incorporated into nanoliposomes/nanoliposomal gel	2% H. sabdariffa nanoliposome gel	In vivo: skin-aging model with topical delivery	20 weeks as reported in the study extraction; confirm exact schedule in the final proof	HPLC/ESI-PDA-MS profiling reported phenolic metabolites, flavonoids and hibiscus acid	Healthy non-induced control; aged/model control; 2% green-tea nanoliposome comparator
Nasser E.J.M. et al., 2025 [27]	Flowers	Ethanolic extraction	Crude ethanolic flower extract	Concentration series 1.96–1,000 µg/mL across assays	In vitro: human keloid fibroblasts; normal fibroblasts used for	Overnight for apoptosis assay; 24 h for normal-fibroblast	Polyphenols/anthocyanins described broadly; detailed quantitative profile not reported in the review extraction	Untreated/model controls; dexamethasone comparator as reported

					cytotoxicity comparison	cytotoxicity assay		
Shadhan, R.M.; Bohari et al., 2017 [26]	Whole fruit	Methanol: water (4:1) maceration for 96 h; partitioned into n-hexane, ethyl acetate and butanol fractions	Crude extract and solvent fractions	Scratch-assay concentration should be taken from the original wound-assay protocol; 6.25–50 mg/mL applies to the glucose-diffusion assay and must not be transferred to wound healing	In vitro: HSF 1184 human skin fibroblast scratch assay	Scratch-wound response assessed at 6 h; MTT assay duration reported separately	Detailed individual compound profile not reported; solvent fractions were prepared	Distilled water/vehicle and assay-specific comparators; acarbose relates to glucose-diffusion assay, not wound healing
Rambe et al., 2022 [25]	Leaves	Ethanolic extraction (96% ethanol) followed by concentration; gel formulation	Leaf-extract gel	5%, 10% and 15% gel	In vivo: rat excision-wound model; topical	Twice daily for 14 days; wound assessment on days 1, 7 and 14	Qualitative phytochemical screening reported; compounds from prior literature should not be represented as quantitatively measured in this study	Gel base; Bioplacenton® comparator
Wang et al., 2022 [24]	Calyces	Aqueous and ethanolic extracts; ethanol extract fractionated chromatographically; hibiscus acid isolated	Aqueous/ethanolic extracts, fractions and isolated hibiscus acid	Assay-specific concentrations for each extract/fraction/hibiscus acid; hibiscus acid purity reported as 99.7%	In vitro: dermal fibroblasts, HaCaT keratinocytes and RAW 264.7 macrophages	Assay-specific (reported across cellular experiments)	Hibiscus acid; broader extract/fraction profiling included flavonoids, polyphenols and anthocyanins	Untreated and LPS-stimulated/model controls; quercetin comparator in NO assay where reported

Note: "Assay-specific" indicates that the dose or exposure conditions varied across experimental endpoints; therefore, a single pooled value would not accurately represent the intervention conditions. "Not reported" indicates that the information was not explicitly provided in the original article. For Ticona et al. (2022) [15], the original article identifies the plant material as leaves and the extraction solvent as water. For Khalil et al. (2022) [31], the original article reports the use of flowers extracted with 70% ethanol, formulated as a 10% (w/w) ointment, and administered topically three times daily, with tissue collection at 36 h. Dose and duration information reported for separate assays, such as the glucose-diffusion assay, should not be extrapolated to skin-related endpoints unless the original article explicitly establishes that they apply.

Table S4. Cross-tabulation of preparation types and dermatological outcome domains investigated in the included preclinical studies.

Study	Specific preparation	Inflammation	Oxidative stress	Wound healing / tissue regeneration	Anti-melanogenic	Anti-fibrotic / photoaging
Ticona et al., 2022 [15]	Aqueous leaf extract (AEHS)	✓	—	—	✓	—
Azizah et al., 2020 [35]	Calyx extract; oral/topical	—	—	✓	—	—
Builders P.F. et al., 2013 [36]	Methanolic calyx-extract creams	—	✓	✓	—	—
Chiu C.T. et al., 2015 [37]	Polyphenolic leaf extract (HLP)	—	—	—	✓	—
Cho et al., 2023 [17]	Hot-water plant extract (HSPE) and callus extract (HSCE)	✓	✓	—	✓	—
Kim et al., 2024 [23]	Free and liposome-encapsulated anthocyanins	—	—	—	✓	—
Hwang J.M. et al., 2013 [33]	70% ethanol flower extract; 10% ointment	✓	✓	✓	—	—
Putra et al., 2020 [32]	Plant-cell-derived Gly-Pro dipeptide	—	—	—	—	✓
Khalil et al., 2022 [31]	Callus-derived HSC-PDRN	—	—	✓	—	—
Kim H.E. et al., 2023 [22]	Aqueous calyx extract (HSCAE)	—	✓	—	✓	✓
Li J. et al., 2020 [34]	Ethanolic calyx extract	✓	✓	—	—	—
Malar D.S. et al., 2022 [30]	Nanoliposomal extract gel	—	✓	—	—	✓
Nasser E.J.M. et al., 2025 [27]	Crude ethanolic flower extract	—	—	—	—	✓
Shadhan, R.M.; Bohari et al., 2017 [26]	Fruit extract and solvent fractions	—	—	✓	—	—
Rambe et al., 2022 [25]	Ethanolic leaf-extract gel	—	—	✓	—	—

Wang et al., 2022 [24]	Aqueous/ethanolic extracts, fractions and hibiscus acid	✓	✓	—	—	✓

Note: A check mark (✓) indicates that a study investigated the corresponding outcome domain, whereas an em dash (—) indicates that the domain was not mapped as a primary outcome in this table. These symbols represent outcome coverage and should not be interpreted as evidence of statistical significance, effect direction, or certainty of evidence. Outcome domains were mapped conservatively, recognizing that some studies investigated overlapping endpoints related to antioxidant activity, inflammation, photoaging, and tissue regeneration.

Supplementary Table S5. Safety and translational characteristics of *Hibiscus sabdariffa* L. preparations in the included preclinical studies

Author (year)	Cell viability/cytotoxicity	Adverse effects/toxicity	LD_{50}	Bioavailability/skin penetration	Pharmacokinetics/metabolites
Ticona et al., 2022 [15]	LDH-based cytotoxicity assay in RAW 264.7 macrophages and B16F10 murine melanoma cells. CC_{50}: 90.69 µg/mL (RAW 264.7) and 81.96 µg/mL (B16F10). These are cell-line-specific in-vitro values, not systemic toxicity thresholds.	No dedicated in-vivo adverse-effect or systemic-toxicity assessment was reported in this in-vitro study. Cytotoxicity was measured in the two cell lines described.	No LD_{50} reported; CC_{50} values are reported for cell cultures and must not be relabelled as LD_{50}.	Aqueous leaf extract was tested in cell culture. Skin penetration and in-vivo bioavailability were not investigated in this study.	No pharmacokinetic or in-vivo metabolite-disposition study reported. ^{1}H-NMR profiling identified phenolic acids, flavonoids, and anthocyanins; chemical profiling is not pharmacokinetics.
Azizah et al., 2020 [35]	Not applicable as a cell-culture cytotoxicity endpoint; the study used a rat third-degree-burn model (n=30) and measured re-epithelialization.	No dedicated systemic toxicity, local irritation, or adverse-event endpoint was reported in the described methods/results. The study assessed wound histology and re-epithelialization, not a formal safety profile.	No LD_{50} reported in the article.	Oral and topical calyx-extract administration were evaluated in rats (250 mg/kg as described by the authors). Quantitative skin penetration, systemic exposure, and bioavailability were not measured.	No pharmacokinetic or metabolite-disposition analysis reported.
Builders P.F. et al., 2013 [36]	No cell-viability/cytotoxicity assay reported; the study evaluated formulated calyx methanol extract in a rat excision-wound model.	No dedicated extract-specific adverse-effect or systemic-toxicity assessment reported. Cream appearance,	No LD_{50} reported in the article.	Topical water-in-oil creams containing 1%, 5%, or 10% w/w calyx extract were applied to rat wounds. Skin penetration and systemic	No pharmacokinetic or metabolite-disposition analysis reported. TLC fingerprinting and total

		consistency, homogeneity, pH, and short-term formulation stability were evaluated; these are formulation-quality measures, not evidence of biological safety.		bioavailability were not measured.	flavonoid/antioxidant analyses were performed.
Chiu C.T. et al., 2015 [37]	Trypan-blue cell-viability assay after 24 h in human A375 melanoma cells, murine B16F10 melanoma cells, and normal human skin fibroblasts (WS1). HLP inhibited melanoma-cell growth dose-dependently; A375 IC_{50} was approximately 250 μg/mL. WS1 viability did not change under the tested conditions.	No in-vivo adverse-effect or systemic-toxicity assessment reported. The observed cell death is an intended anticancer effect in melanoma cells; normal-fibroblast findings were limited to the in-vitro assay.	No LD_{50} reported in the article.	In-vitro cell exposure only. Skin penetration and in-vivo bioavailability were not investigated.	No pharmacokinetic or in-vivo metabolite-disposition analysis reported. The extract was chemically profiled for polyphenols, including epicatechin gallate; this is compositional analysis, not pharmacokinetics.
Cho et al., 2023 [17]	CCK-8 assay in HaCaT cells: HSCE cell viability ranged from 85.56% to 76.16% as extract concentration increased from 1% to 10% (Figure 3A, p. 5). The authors later describe no cytotoxicity under their tested conditions; report the numerical values alongside that interpretation.	No dedicated in-vivo adverse-event, irritation, or systemic-toxicity study was described; evidence is limited to in-vitro human skin-cell assays.	No LD_{50} reported; the study did not report an acute-lethality experiment.	Plant and callus water extracts were tested in cultured cells. Direct skin penetration and in-vivo bioavailability were not assessed.	HPLC identified several extract components, including guanine, phenylalanine, and tryptophan in both extracts, and adenine, uridine, adenosine, and guanosine in HSCE. This was compositional profiling, not pharmacokinetic or in-vivo metabolite-disposition analysis.
Kim et al., 2024 [23]	MTT assay in human A375 melanoma cells after 72 h: liposome-encapsulated	Safety evidence is limited to the 72-h A375 cell-viability assay; no in-vivo	No LD_{50} reported; the reported concentrations are	The study characterized liposomes and examined anthocyanin release in PBS in	In-vitro release kinetics of liposome-encapsulated anthocyanin were studied in

	anthocyanin (LCA) at 5–50 mg/mL showed no significant cytotoxic effect; viability decreased by approximately 20% at 200 mg/mL (Results, 'Effects of LCA on cell viability').	adverse effects or systemic toxicity were evaluated in this study.	from an in-vitro cell assay.	vitro. It did not measure skin penetration or in-vivo bioavailability.	PBS. No pharmacokinetic study or in-vivo metabolite-disposition analysis was reported.
Hwang J.M. et al., 2013 [33]	No cell-culture viability assay; the study evaluated a topical 10% (w/w) flower-extract ointment in a rat burn model.	The authors describe an acute dermal irritation/toxicity assessment in rats (n = 6) and monitored erythema, irritation, inflammation, and itching against ointment-base controls. The methods state the preparation was considered suitable for use; numerical scores or a detailed adverse-event table were not identified in the extracted report. This is a local dermal assessment, not a comprehensive systemic safety study.	No LD_{50} value reported. The described topical assessment should not be interpreted as an LD_{50} determination.	Topical ointment was applied to rat skin/burn wounds. No quantitative skin-penetration or systemic bioavailability measurement was reported in the article.	No pharmacokinetic or metabolite-disposition study reported.
Putra et al., 2020 [32]	The study tested plant-cell-derived Gly–Pro (GP) in human dermal fibroblasts for antifibrotic effects. A dedicated cell-viability/cytotoxicity assay was not identified in the reported methods/results;	No dedicated in-vivo adverse-effect or systemic-toxicity assessment reported; experiments were conducted in cell models.	No LD_{50} reported.	No skin-penetration or in-vivo bioavailability assessment reported.	LC–MS/MS was used to identify/quantify dipeptides in the plant-cell extract. This is compositional analysis, not pharmacokinetics or in-vivo metabolite disposition.

	efficacy findings should not be interpreted as evidence of safety.				
Khalil et al., 2022 [31]	CCK-8 assay in HaCaT keratinocytes after 24 h at 1, 2, 5, and 10 µg/mL HSC-PDRN. The 5 µg/mL treatment gave the highest viability: 126.08% of control in HaCaT cells. In Detroit 551 dermal fibroblasts, viability was 148.06% at 5 µg/mL and 134.07% at 10 µg/mL (Results, Figure 1g). These are proliferation/viability outcomes, not an LD_{50}.	No in-vivo adverse-event, irritation, or systemic-toxicity assessment was described; evidence is limited to in-vitro cell assays.	No LD_{50} reported; this was an in-vitro PDRN study.	HSC-PDRN was applied directly to cultured skin cells. Skin penetration and in-vivo bioavailability were not measured.	No pharmacokinetic or in-vivo metabolite-disposition study was reported. The study assessed cellular and molecular wound-healing responses to plant-callus-derived PDRN.
Kim H.E. et al., 2023 [22]	MTT assay in HS68 human skin fibroblasts after 24 h: aqueous calyx extract at 0.1–2 mg/mL showed no apparent toxicity and increased proliferation; concentrations above 2 mg/mL caused severe toxicity, with approximately 80% cell death reported. The authors linked high-concentration toxicity partly to the extract's low pH; pH adjustment reduced the toxic effect (Fig. 1, p. 5 of article).	Cell-culture cytotoxicity was assessed. The study did not constitute an in-vivo adverse-effect or systemic-toxicity assessment.	No LD_{50} reported.	The study evaluated an aqueous extract in cell culture; quantitative skin penetration and in-vivo bioavailability were not assessed.	No pharmacokinetic or in-vivo metabolite-disposition study reported.

Li J. et al., 2020 [34]	MTT assay in HaCaT cells after 24 h: calyx ethanol extract at 20–100 μg/mL did not exhibit detectable toxicity in the tested range. Phenanthrene at 250 μM produced approximately 50% cell death. Extract pretreatment improved viability after phenanthrene exposure, with the strongest trend up to 60 μg/mL and a weaker response at 80–100 μg/mL; extract alone at 100 μg/mL had viability similar to control (Fig. 1, p. 6).	Cellular protection against phenanthrene-induced toxicity was investigated; systemic, dermal-irritation, or long-term safety was not assessed by this in-vitro study.	No LD_{50} reported.	Skin penetration and in-vivo bioavailability were not assessed.	Expression of phase I/II metabolism-related genes and CYP1A1/CAR/PXR/RXR-associated mechanisms was investigated. These findings do not establish whole-organism pharmacokinetics or metabolite disposition.
Malar D.S. et al., 2022 [30]	The study used an in-vivo UV/galactosamine-induced skin-aging model in mice; a cell-culture viability/cytotoxicity assay was not reported in the described methods/results. This should not be interpreted as evidence of cellular safety.	The study reports efficacy and histopathological outcomes in the skin-aging model, but does not describe a dedicated adverse-event, local-irritation, or systemic-toxicity assessment. Safety conclusions should therefore remain limited.	No LD_{50} value or acute-lethality experiment reported.	Confocal microscopy showed deep dermal localization/penetration of H. sabdariffa-loaded nanoliposomes (approximately 400 nm) in skin. This supports local skin penetration in the experimental model, but does not establish systemic bioavailability.	HPLC/ESI/PDA/MS^n profiling identified phenolic metabolites in the extract. This is chemical characterization; the study did not report systemic pharmacokinetics or in-vivo disposition of individual metabolites.
Nasser E.J.M. et al., 2025 [27]	Normal human skin fibroblast cytotoxicity/viability assay after 24 h across 1.96–1000 μg/mL ethanolic flower extract. Reported viability	In-vitro normal-fibroblast viability was assessed; no in-vivo adverse effects or systemic toxicity were evaluated.	No LD_{50} reported; the study tested cultured cells, not acute lethality in animals.	Extract was tested directly in cultured keloid and normal fibroblast cells. Skin penetration and in-vivo bioavailability were not assessed.	No pharmacokinetic or in-vivo metabolite-disposition analysis was reported.

	ranged from 89.0% to 99.2%, with no significant differences in absorbance/viability across concentrations (Table 1, p. 3).				
Shadhan, R.M.; Bohari et al., 2017 [26]	No cell-culture viability/cytotoxicity assay reported. The study evaluated topical 5%, 10%, and 15% ethanolic roselle-leaf gel in a rat excision-wound model and measured wound size and epithelial thickness.	The reported endpoints were wound healing and histology over 14 days. A dedicated adverse-event, irritation, or systemic-toxicity assessment was not described; wound-healing outcomes alone do not establish safety.	No LD_{50} value or acute-lethality experiment reported.	Topical gel was applied to rat excision wounds. Quantitative skin penetration and systemic bioavailability were not measured.	No pharmacokinetic or in-vivo metabolite-disposition analysis reported.
Rambe et al., 2022 [25]	MTT assay in human skin fibroblasts (HSF 1184) after treatment with fruit methanolic extract and solvent fractions. The authors reported IC_{50} values of 79 mg/mL for methanolic extract, 79 mg/mL for ethyl-acetate fraction, 125 mg/mL for n-hexane fraction, and 112 mg/mL for butanol fraction (Results, section 3.3 and Table 1, pp. 468–469). These are in-vitro cell-viability values, not LD_{50} values.	Safety evidence was limited to in-vitro fibroblast cytotoxicity testing. No in-vivo adverse-effect or systemic-toxicity assessment was reported.	No LD_{50} value reported; IC_{50} values were measured in cultured fibroblasts.	In-vitro cell exposure only; skin penetration and in-vivo bioavailability were not investigated.	No pharmacokinetic or in-vivo metabolite-disposition analysis reported. The study used methanolic fruit extract and solvent fractions for enzyme, glucose-diffusion, and scratch-assay experiments.
Wang et al., 2022 [24]	MTT assay showed that crude aqueous and ethanolic	Cell-based cytotoxicity was assessed, including	No LD_{50} value reported; the	Skin penetration and systemic bioavailability were not	The study performed bioactivity-guided

	calyx extracts were not cytotoxic to NHDF-Ad skin fibroblasts at concentrations up to 1,000 μg/mL (Results, section 2.1, p. 2). The study also tested fractions and isolated hibiscus acid in cell models; do not generalize the crude-extract result to every fraction or compound.	additional cellular experiments in skin fibroblasts and RAW264.7 macrophages. No in-vivo adverse-event, irritation, or systemic-toxicity assessment was reported.	safety-related findings are from cell assays, not acute-lethality testing.	assessed; materials were tested directly in cultured cells.	fractionation and chemical identification of hibiscus acid and other extract constituents. These are compound-identification results, not pharmacokinetics or in-vivo metabolite-disposition data.

Abbreviations. LD_{50}, median lethal dose; HaCaT, human keratinocyte cell line; A375 and B16F10/B16F1, melanoma cell lines; NHDF-Ad, adult human dermal fibroblasts; RAW264.7, murine macrophage cell line; HSC-PDRN, H. sabdariffa callus-derived polydeoxyribonucleotide.
